# Global Research Trends in Tendon Stem Cells from 1991 to 2020: A Bibliometric and Visualized Study

**DOI:** 10.1155/2022/7937765

**Published:** 2022-06-18

**Authors:** Huibin Long, Ziyang Yuan, Heyong Yin, Bo Yang, Ai Guo

**Affiliations:** Department of Orthopedics, Beijing Friendship Hospital, Capital Medical University, Beijing, China

## Abstract

**Background:**

Tendinopathy is a disabling musculoskeletal disorder affecting the athletics and general populations. There have been increased studies using stem cells in treating tendon diseases. The aim of this bibliometric and visualized study is to comprehensively investigate the current status and global trends of research in tendon stem cells.

**Methods:**

Publications related to tendon stem cells from 1991 to 2020 were retrieved from Web of Science and then indexed using a bibliometric methodology. VOSviewer software was used to conduct the visualized study, including coauthorship, cocitation, and cooccurrence analysis and to analyze the publication trends of research in tendon stem cells.

**Results:**

In total, 2492 articles were included and the number of publications increased annually worldwide. The United States made the largest contribution to this field, with the most publications (938 papers, 37.64%), citation frequency (68,195 times), and the highest *H*-index (103). The most contributive institutions were University of Pittsburgh (96 papers), Zhejiang University (70 papers), Shanghai Jiao Tong University, and Chinese University of Hong Kong (both 64 papers). The *Journal of Orthopaedic Research* published the most relative articles. Studies could be classified into five clusters: “Animal study,” “Tissue engineering,” “Clinical study,” “Mechanism research,” and “Stem cells research”, which show a balanced development trend.

**Conclusion:**

Publications on tendon stem cells may reached a platform based on current global trends. According to the inherent changes of hotspots in each cluster and the possibilities of cross-research, the research in tendon stem cells may exist a balanced development trend.

## 1. Introduction

As an essential connection and force transmission between muscle and bone, tendons play roles in storing elastic energy and withstanding high tensile forces that enable movements [1]. Tendinopathy or chronic tendon injury, characterized by pain and impaired performance, is commonly caused by overuse [2]. Population aging and increased sports participation have made tendon injuries common in clinical medicine [3]. Certain tendons, such as the rotator cuff, Achilles, tibialis posterior, and patellar tendons, are suggested to be more likely to pathology, for whose pathological damages are often based on the process of degeneration. Additionally, the extensor and flexor tendons of the hands and fingers often directly suffered from laceration at all ages [4]. Approximately 30% of general musculoskeletal consultations are associated to tendinopathy [5]. Moreover, it is estimated that the United States spent $30 billion on musculoskeletal disorders each year, and tendon/ligament injuries account for almost 45% of the expenditure [6, 7].

Surgical techniques are the most commonly used strategy to repair ruptured tendons, but the incomplete healing of the tendon through surgical procedure happens on occasion. Moreover, the long-term effect of surgical techniques is poor especially among the elderly due to rerupture, restrictive adhesions, poor strength, and function related to reduced biomechanical properties [8]. Hence, nonsurgical treatment, compared to the traditional surgical treatment with limitations and complications, has attracted people's attention in recent years.

The primary target of tendinopathy treatment is to alleviate pain and reduce the inflammation and swelling of the injured tendons. Nonsurgical treatment such as rest, cryotherapy, nonsteroidal anti-inflammatory drugs, injection with corticosteroids, continuous passive motion, and restrictive bracing, have been well recognized as effective ways for tendon injuries [9–11]. However, corticosteroids, as well as nonsteroidal anti-inflammatory drugs, can only alleviate pain and inflammation but take no effect on tendon healing [12–14].

Cell-based tissue engineering has been attractive and widely explored for musculoskeletal regeneration. In 2003, Smith et al. [15] implanted autologous bone marrow-derived mesenchymal stem cells (MSCs) into superficial digital flexor tendon of horse that had suffered a strain-induced injury, and the results revealed positive influence. Since Bi et al. [16] first demonstrated the existence of stem cells in tendon tissue at 2007, various studies have focused on the isolation, characterization, and finding of specific markers of tendon stem cells [17, 18]. Owning potential to differentiate into tenocytes, a high proliferative and synthetic activity, the secretion of paracrine factors and the ability to exhibit immunomodulatory effects, tendon stem cells are promising to promote tendon regeneration [19]. And a series of studies have reported the effectiveness of stem cells for tendon healing [20–22], inspiring further researches to investigate the underling mechanism.

Publication is a critical part of scientific research and regarded as a significant indicator of research contribution [23]. Based on the literature databases and literature metrology characteristics, bibliometric analysis not only provides information about the development of a certain field and comparison of contributions among institutions, countries, and journals but also evaluates trends in research activity over time [24–26] and has been applied in developing policy and clinical practice guidelines [27].

Nevertheless, the global development trend regarding tendon stem cells has not been well studied yet. To promote better development in tendon stem cells, we feel it necessary to summarize the current status and provide an overview of research trends in this field.

## 2. Materials and Methods

### 2.1. Data Source

Bibliometric analysis was performed based on the Science Citation Index-Expanded (SCI-E) and Social Sciences Citation Index (SSCI) of the Web of Science (WoS), which are considered prominent databases for bibliometrics, covering more than 12,000 international scientific journals with the most impact and the highest quality [28].

### 2.2. Search Strategy

All publications were searched in WoS from database inception to 31 December, 2020, which included the articles in this field over the past 30 years. In the present study, the search terms were as follows: Topic = stem cell AND Topic = tendon AND Year Published = (1991–2020) AND Language = (English) AND Document types = (Article OR Review). We also refined the search for certain countries or regions by indexing country/region in the WoS. A retrieval work was performed in the same day (on September 25, 2021) to avoid the variations due to daily updates. Informed consent was not required because the data are all secondary data without any personal information.

### 2.3. Data Collection

The full records of all eligible publications, including title, authors, year of publication, nationalities, affiliations, journal, keywords and abstract, were downloaded as TXT files from the WoS database. Two authors (HB Long and ZY Yuan) independently screened and extracted the data entry and collection from the search results, to make sure no papers were misclassified. Any disagreement was resolved by discussion for reaching a consensus. Finally, the two authors manually verified and inputted the data in Microsoft Excel 2019 and further analyzed the data with GraphPad Prism 9 and VOSviewer separately.

### 2.4. Bibliometric Analysis

The basic characteristics of publications (including authors, year of publication, nationalities, institutions, journal, research orientation, times cited, and *H*-index) were described using the intrinsic function of WoS. *H*-index is a useful measure to evaluate the impact of a scientific study and shows that a scientist or scholar has published *H* papers, each of which has been cited in other publications at least *H* times. As a result, the *H*-index reflects both the number of publications and the number of citations per publication [29, 30]. R software (version 4.0.2, R core team) was used to draw the time curve of publications using a logistic regression model: *f* (*x*) = *c*/[1 + *a* × exp^(−*b* × (*x* − 1991))^]. In this formula, the independent variable *x* refers to the year and the *f*(*x*) refers to the corresponding number of publications. The formula *T* = Lna/*b* + 1991 was used to calculate the inflection point defined as the time when the growth rate of publications changes from positive to negative.

### 2.5. Visualized Analysis

VOSviewer (Leiden University, Leiden, The Netherlands) was used for the visualized analysis of the publications [28, 31]. In the present study, VOSviewer was used for coauthorship, cocitation, and cooccurrence analyses.

## 3. Results

### 3.1. Global Publication

#### 3.1.1. Number of Global Publications

**Figure 1:**
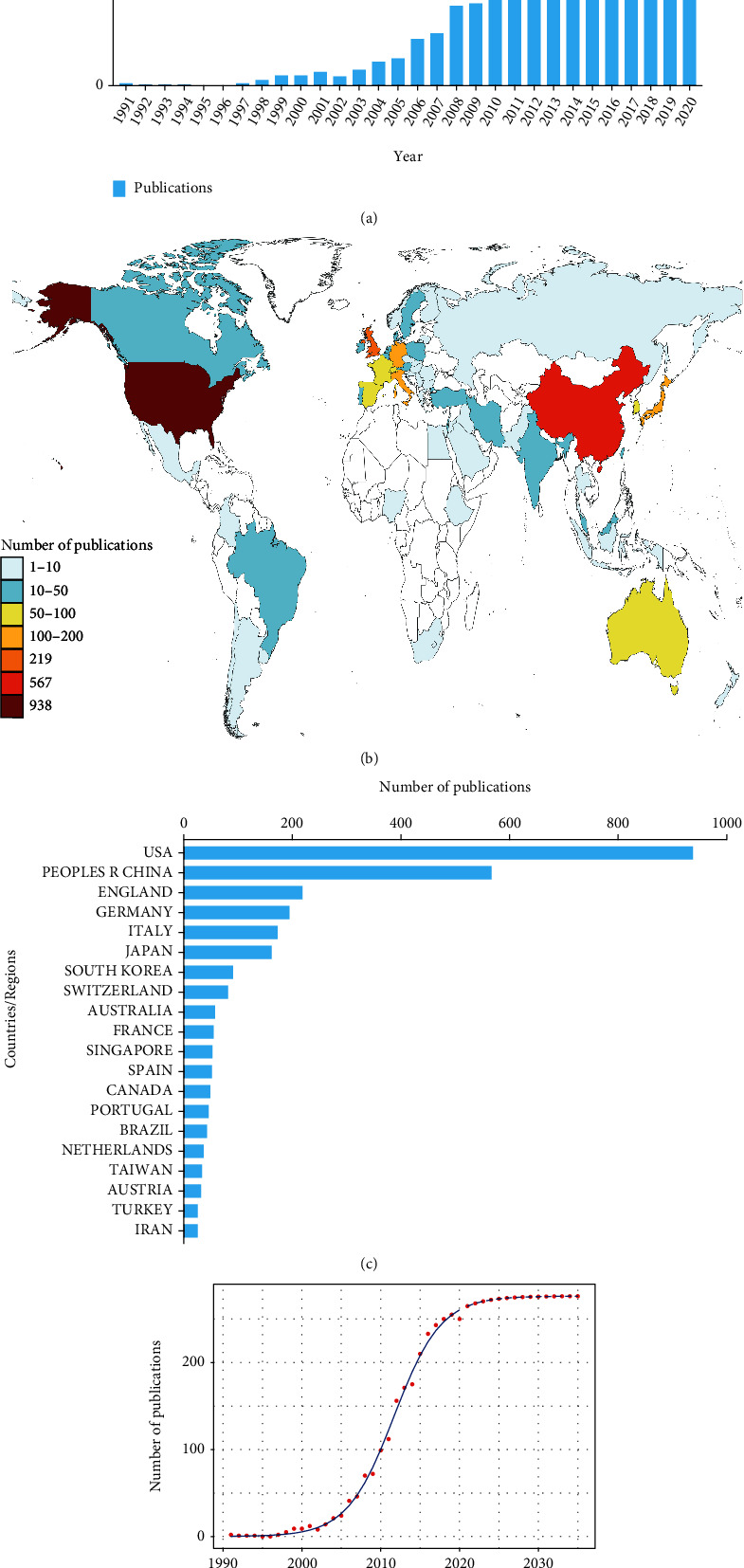
(a) The total number of publications related to the publications of tendon stem cells. The blue bars indicate the number of publications each year. (b) Distribution of the publications of tendon stem cells in a world map. (c) The top 20 countries of total number of publications. (d) Model fitting curves of trends in global publications.

In total, 2492 publications met the criteria. From 1991 to 2020, there has been a steadily increasing trend of global publications annually. The number of publications increased from 2 (1991) to 250 (2020). Most research was published in 2019 (255, 10.23%) (Figure 1(a)).

#### 3.1.2. Contributions of Countries and Regions

A total of 65 countries and regions published articles in this domain. The United States published the most papers (938, 37.64%), followed by China (567, 22.75%), England (219, 8.79%), and Germany (195, 7.83%). (Supplementary Table 1; Figures 1(b) and 1(c)).

#### 3.1.3. Global Trends of Publications

The logistic regression model was used to construct the time curve of the number of publications and helped to predict the future trend in the next few years.  Figure 1(d) shows the model fitting curves of the growth trend. According to the time curve, the number of publications in this field was estimated to reach a platform.

### 3.2. Scientific Impact of the Publications

#### 3.2.1. Countries and Regions

**Figure 2:**
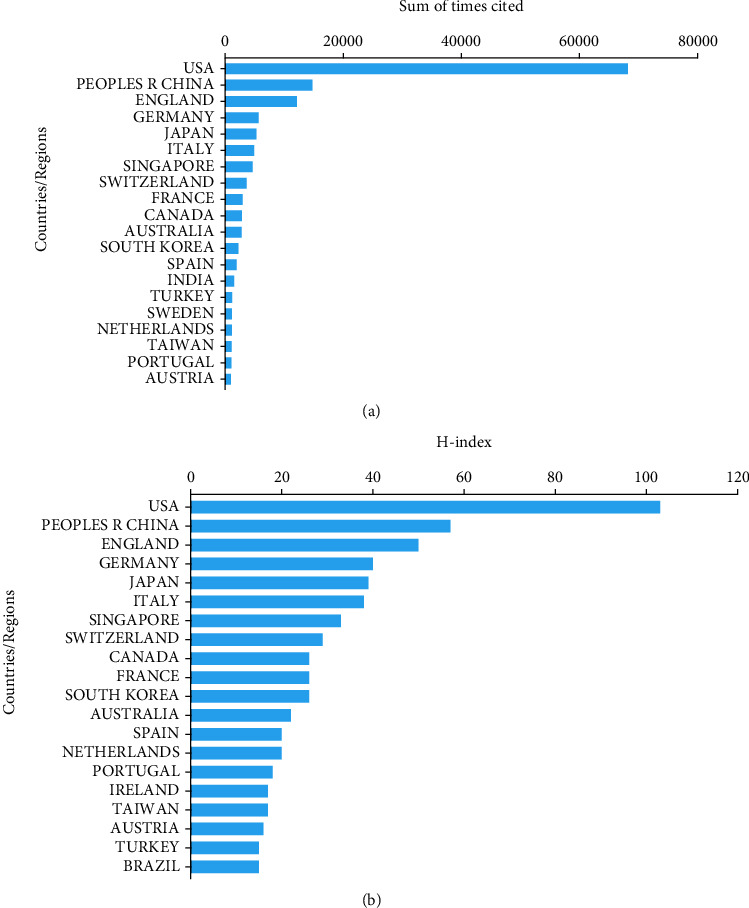
(a) The top 20 countries of total citations. (b) The top 20 countries of *H*-index of publications.

Papers from the United States had the highest citation frequency (68,195). Then, China ranked second in total citation frequency (14,776), followed by England (12,132), Germany (5,657), and Japan (5,266) (Figure 2(a)).  Figure 2(b) shows the *H*-index of top 20 countries. The United States outranked other countries with *H*-index of 103, followed by China (57), England (50), Germany (40), and Japan (39).

#### 3.2.2. Journals

**Figure 3:**
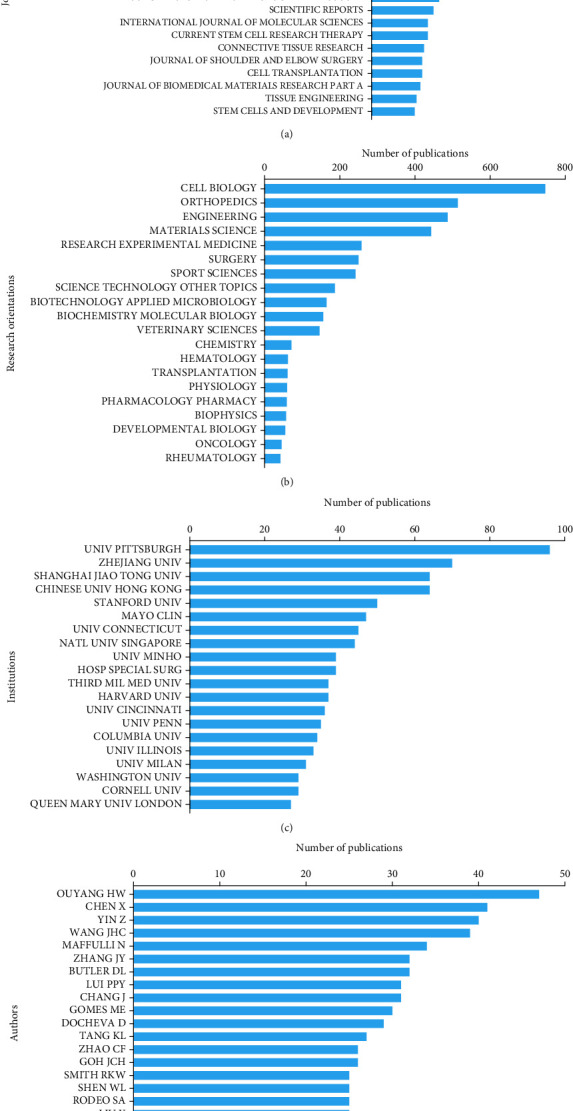
(a) The top 20 journals with most publications related to the research of tendon stem cells. (b) The 20 research orientations and the number of publications in each orientation. (c) The top 20 institutions of high-impact and the number of their publications. (d) The top 20 authors with most publications in this field.

The *Journal of Orthopaedic Research* published the most articles, with 100 publications. *American Journal of Sports Medicine* ranked second with 83 articles. Moreover, there were 77 articles in *Tissue Engineering Part A* and 63 articles in *Biomaterials* on the research of tendon stem cells.  Figure 3(a) shows the top 20 journals with most papers, and Supplementary Table 2 shows all journals and number of papers published in each.

#### 3.2.3. Research Orientations

Figure 3(b) shows the top 20 research orientations related to tendon stem cells. The top 5 popular areas of research were cell biology (747 papers), orthopedics (514 papers), engineering (487 papers), materials science (443 papers), and research experimental medicine (258 papers), respectively.

#### 3.2.4. Institutions

The top 20 contributive institutions with the most articles are shown in Figure 3(c). The University of Pittsburgh published the most articles, with 96 papers. Zhejiang University ranked second (70 papers), followed by Shanghai Jiao Tong University and Chinese University of Hong Kong (both 64 papers). All institutions and number of papers published in each are shown in Supplementary Table 3.

#### 3.2.5. Authors

The top 20 authors published a total of 637 papers, which accounted for 25.56% of all articles in this field (Figure 3(d)). Ouyang HW from China published the most researches on tendon stem cells with 47 papers, followed by Chen X with 41 papers and Yin Z with 40 papers. In this study, we included all authors into the analysis, regardless of the relative contributions of the authors to a single study.

### 3.3. Coauthorship Analysis

Coauthorship analysis presents the relationship of items which was built according to the number of coauthored documents, which acts as an effective role in evaluating leading scientists, countries, and organizations [32].

#### 3.3.1. Countries and Regions

**Figure 4:**
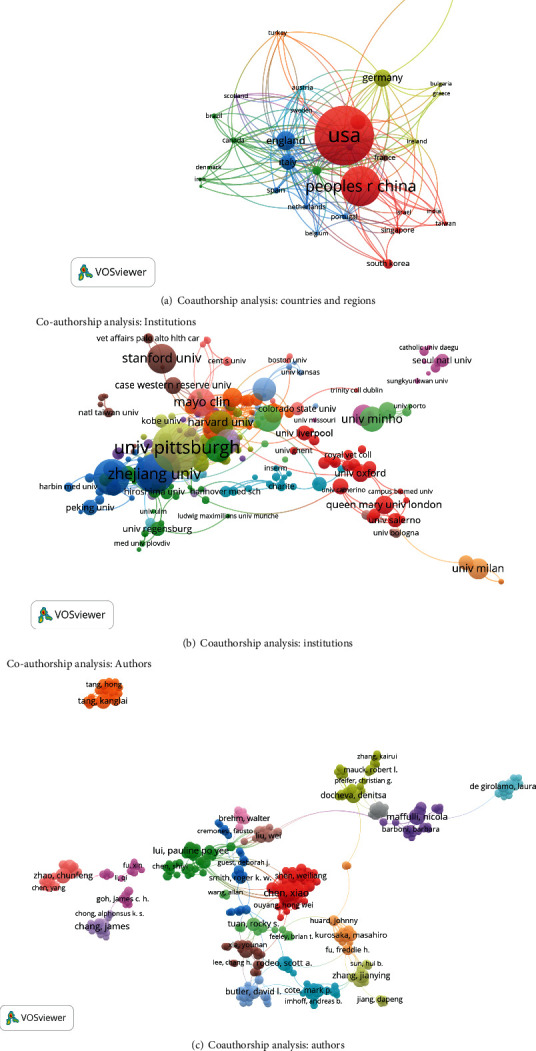
Coauthorship analysis of global research of tendon stem cells. (a) Mapping on the 32 identified countries by co-authorship analysis in this field. (b) Mapping on the 265 identified institutions by coauthorship analysis in this field. (c) Mapping on the 378 identified authors by coauthorship analysis on the research of stem cells for tendon.

The studies in 32 identified countries and regions (the minimum number of studies from a country or region was over five) were analyzed by VOSviewer (Figure 4(a)). The top five countries and regions with the greatest total link strengths were as follows: United States (337 times), China (210 times), England (152 times), Germany (140 times), and Italy (104 times).

#### 3.3.2. Institutions

Studies (defined as minimum number of documents form an organization that were used more than five) identified in the 265 institutions were analyzed by VOSviewer (Figure 4(b)). The top five institutions with the greatest total link strengths were as follows: Chinese University of Hong Kong (86 times), Zhejiang University (81 times), Harvard University (73 times), University of Pittsburgh (70 times), and Columbia University (66 times).

#### 3.3.3. Authors

378 authors were identified (the minimum number of publications from an author was over five), and their publications were analyzed by VOSviewer (Figure 4(c)). The top five authors with the greatest total link strengths were as follows: Chen X (262 times), Yin Z (245 times), Heng BC (181 times), Shen WL (129 times), and Tang KL (122 times).

### 3.4. Cocitation Analysis

Cocitation analysis indicates the relationship of items which are built based upon the number of times they were cited together, and it has abilities to avoid academic isolation and to accelerate knowledge integration for consistency of cross discipline [33].

#### 3.4.1. Journals

**Figure 5:**
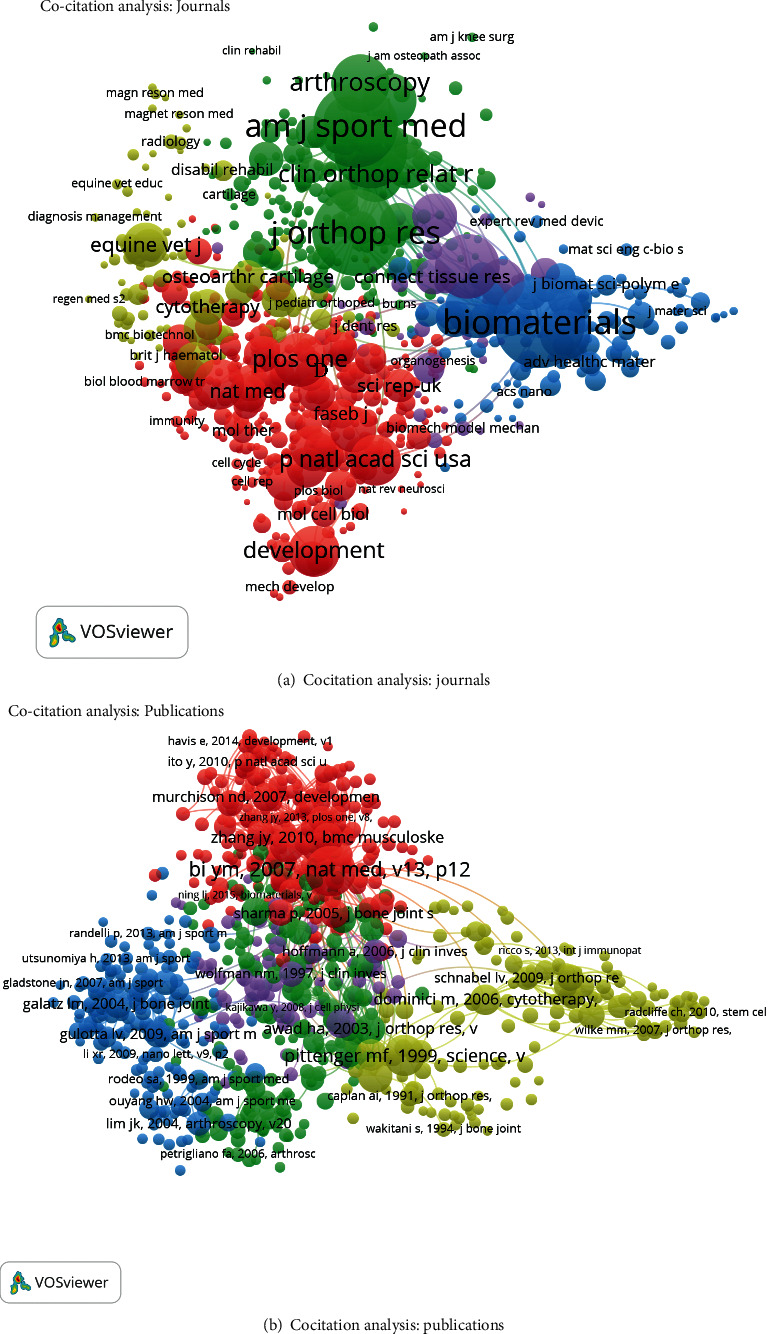
Cocitation analysis of global research of tendon stem cells. (a) Mapping on the 825 identified journals by cocitation analysis. (b) Mapping on the 808 identified references by cocitation analysis.

The journal was included if the minimum number of citations from a source was over 20. There were 825 journals that met the criteria (Figure 5(a)). The top five journals with the greatest total link strengths were as follows: *Biomaterials* (554,821 times), *Journal of Orthopaedic Research* (429,514 times), *American Journal of Sports Medicine* (385,081 times), *Journal of Bone and Joint Surgery-American volume* (282,060 times), and *Tissue Engineering Part A* (242,487 times).

#### 3.4.2. Publications

A total of 808 publications (the minimum number of citations of a reference was over 20) were analyzed using VOSviewer (Figure 5(b)). The top five publications with the greatest total link strengths were as follows: Bi et al. [16] (9936 times); Schweitzer et al. [34] (5733 times); Pittenger et al. [35] (4522 times); Young et al. [36] (4356 times), and Awad et al. [37] (4314 times).

### 3.5. Cooccurrence Analysis

**Figure 6:**
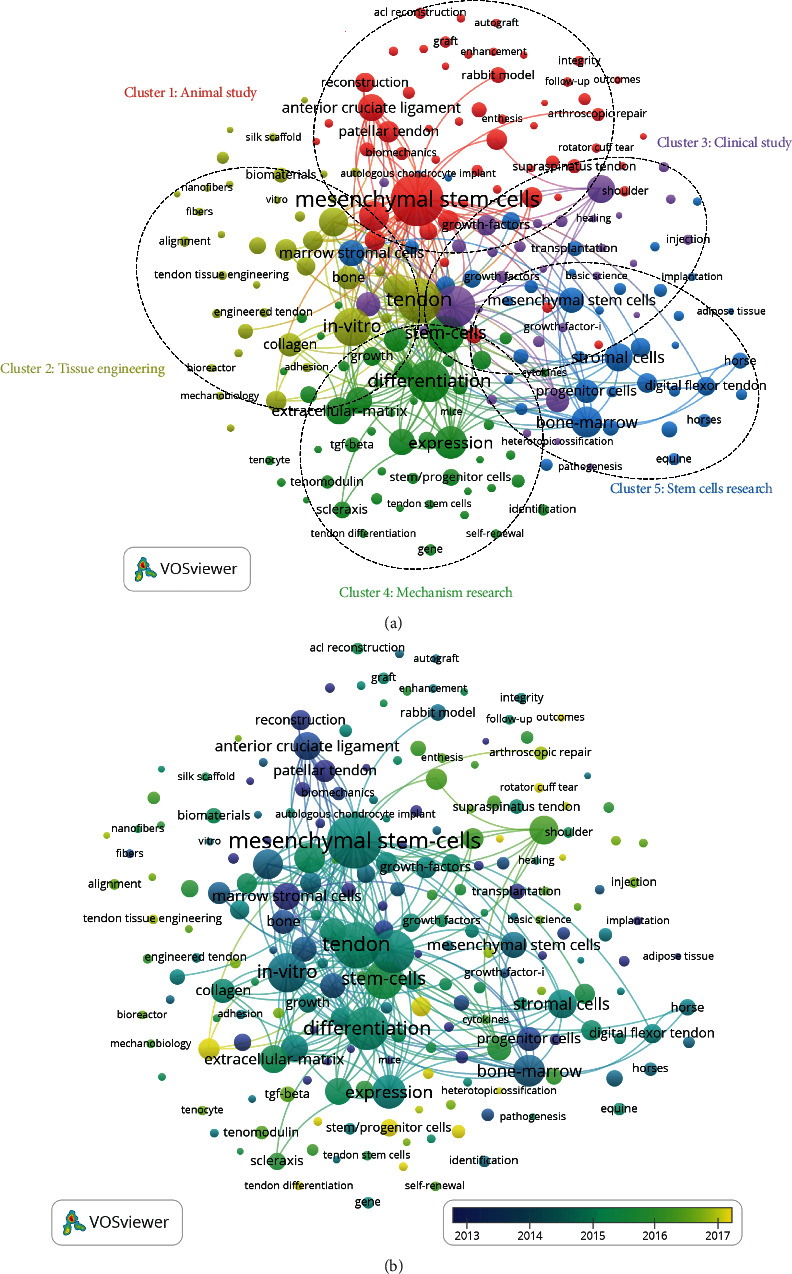
Cooccurrence analysis of global research of tendon stem cells. (a) Mapping on keywords in the research of tendon stem cells; the size of the nodes indicates the frequency, and the keywords are classified into five clusters: animal study (red color), tissue engineering (yellow color), mechanism research (green color), stem cell research (blue color), and clinical study (purple color). (b) Distribution of keywords based upon their time of appearance; purple keywords appeared earlier than green ones, and yellow keywords appear later than both of purple and green keywords.

Cooccurrence analysis identifies that the relationship of items is constructed based upon the number of publications when they occur together. It is a powerful tool to evaluate research areas as well as hot issues and to track the academic progress [38]. The keywords with a minimum number of occurrences over 20 were analyzed by VOSviewer. As illustrated in Supplementary Table 4 and Figure 6(a), the 213 identified keywords could be divided into five clusters: “Animal study,” “Tissue engineering,” “Clinical study,” “Mechanism research,” and “Stem cells research”.

In the “Animal study” cluster, the most used keywords were mesenchymal stem-cells, regeneration, anterior cruciate ligament, and patellar tendon. In the cluster of “Tissue engineering,” the primary keywords were tendon, in-vitro, tissue engineering, and tissue. In the “Clinical study,” the main keywords were repair, platelet-rich plasma, Achilles tendon and tendinopathy. In the “Mechanism research” cluster, the frequently used keywords were differentiation, stem cells, expression and extracellular-matrix. As for the cluster of “Stem cells research,” the main keywords were bone marrow, stromal cells, marrow stromal cells, and mesenchymal stem cells. These results showed the research field distribution of publications related to the study of tendon stem cells.

In Figure 6(b), VOSviewer applied different colors to keywords based on when they appeared in the literature for the average time. The purple color indicated that the keywords appeared earlier while keywords in yellow color appeared later. Generally, each of the five clusters had a trend of balanced development. All five clusters were undergoing different degrees of changes on the research hotspot, which meant a diversified developing trend. Some keywords, such as tenogenic differentiation belonged to the “Tissue engineering” cluster, had potential to be studied more in the future.

## 4. Discussion

### 4.1. Global Trends of Research in Tendon Stem Cells

Bibliometric and visualized research can be used to show the current status and predict the future development trends in research. Thus, the study is aimed at evaluating the status and global trends of tendon stem cells with respect to publications, contributing countries, institutions, and research orientations. As shown in this study, the number of publications increased with time; nevertheless, it might reach a platform in the next few years.

### 4.2. Status and Scientific Impact of Global Publications

The *H*-index and total number of citations represent the academic influence and publication quality of a country [39, 40]. While the United States was the leading contributive country in this field, China was regarded as a vital role which made abundant contribution to this field in terms of total number of publications, total citation frequency, and *H*-index. *Journal of Orthopaedic Research*, *American Journal of Sports Medicine*, *Tissue Engineering Part A*, and *Biomaterials* have great possibilities to propose the latest academic findings in this field (Figure 4(a)). Almost all the top 20 institutions come from the top 5 countries with the largest number of publications, indicating that the academic level of a country mainly depends on the number of its high-quality research institutions. The top 20 authors listed in Figure 3(b), including Ouyang HW, Chen X, and Yin Z, may have a substantial impact on the development and lead the newest advancement in this field.

On the other hand, the association between papers with respect to country, institution, and journal was evaluated by bibliographic coupling analysis in this study. Coauthorship analysis was performed to assess collaboration among countries, institutions, and authors. The one with greater total link strength suggested that the author/institution/country would be more likely to have cooperation with others. In our study, the results of coauthorship analysis were Chen X, Chinese University of Hong Kong, the United States, respectively. Cocitation analysis was conducted to evaluate the academic impact of studies, by counting the number of times cited together. The landmark studies with larger total cocitation frequency could be regarded as the indicator in this field. *Biomaterials* was the journal with the highest citation frequency in this domain.

### 4.3. Research Focus on Tendon Stem Cells

Using the cooccurrence analysis, the research directions and hotspots in this field were identified. The map of the cooccurrence network was constructed based on the keywords of all included studies. As shown in Figure 6(a), five research orientations were discovered, and this result helps establish certain domains for further investigation. In the cooccurrence map, the keywords, including mesenchymal stem-cells, tendon, repair, differentiation, and in-vitro were highlighted with bigger icons. Thus, further high-quality studies concentrating on the mesenchymal stem cells for tendon repair in these five clusters may still be needed to deeply investigate.

The overlay visualization map was color established by VOSviewer, with different colors compared to the map of the cooccurrence network, based on the average time the keywords appeared in the papers [23, 41]. This method was used to monitor the directions of research. In this overlay visualization (Figure 6(b)), the color bar indicated the year of publication. Nevertheless, the density of each color node (from purple to yellow) in the five clusters was evenly distributed, which showed that the five research directions had a balanced development trend. In addition, each direction itself was also undergoing changes in research hotspots, showing a diversified development trend.

Based on the results of our study, the bibliometric and visualized analyses demonstrate encouraging information of stem cells in the treatment of tendon diseases, suggesting that there exist broad research prospects and the feasibility of in-depth research. Using the bibliometric and visualized analysis, researchers can have overall knowledge on the leading countries, authors, and institutions in this field. With the above information, it is easier for investigators to obtain valuable information according to their own requirement. Additionally, based on the cooccurrence analysis and overlay visualization map, researchers could clarify the hot spots and efficiently determine the specific direction of in-depth research; funding agencies could also make more rational investment plan in tendon stem cells.

### 4.4. Strengths and Limitations

The present study provided a comprehensive perspective of the worldwide status and trends of studies about tendon stem cells using bibliometric and visualized analyses. However, there were still a few limitations which are inevitable. In this study, we performed bibliometric analysis based on WoS as database. Other major databases, such as PubMed, Embase, and Cochrane Library, were abandoned, which caused data source bias. Secondly, we only chose English language studies based on WoS; other non-English language papers may have been neglected, especially Chinese. Since China has large population of tendinopathy patients and acts as an important role with huge contribution in the field of research in tendon stem cells, excluding non-English publications may result in language bias. Thirdly, there might be overlapping regarding the coauthorship analysis, considering authors having the same last name and initials, such as “Chen X.” Additionally, some recently published papers with high scientific impact might not be paid enough attention due to the absence of high citation frequency which requires long time to achieve. Therefore, there was still a need to focus on the latest primary studies and other non-English studies in our daily research work.

## 5. Conclusions

This study showed the current status and global trends in tendon stem cells. The United States was the leading country in contribution and influence in this field. The number of publications on stem cells in treating tendon diseases may have reached a platform based on current global trends. Furthermore, there may be a trend of balanced development, with the potential for internal changes in hot spots in various subdirections of the research.

## Data Availability

The datasets used and/or analyzed during the current study are available from the corresponding author on reasonable request.

## Supplementary Materials

Supplementary 1 Supplementary Table 1: countries/regions and number of papers published on tendon stem cells from 1991 to 2020.

Supplementary 2 Supplementary Table 2: journals and number of papers published on tendon stem cells from 1991 to 2020.

Supplementary 3 Supplementary Table 3: institutions and number of papers published on tendon stem cells from 1991 to 2020.

Supplementary 4 Supplementary Table 4: the five clusters of keywords on tendon stem cells research from 1991 to 2020. Keywords with minimum occurrences over 20 were included in the cooccurrence analysis.
