# Supplementary material for: Global Research Trends in Tendon Stem Cells from 1991 to 2020: A Bibliometric and Visualized Study
Source: Stem Cells Int. 2022 Jun 18;2022:7937765. doi: 10.1155/2022/7937765 (PMC9233735; doi:10.1155/2022/7937765)
Supplement: Supplementary 1 — Supplementary Table 1: countries/regions and number of papers published on tendon stem cells from 1991 to 2020. [file 7937765.f1.pdf]

Supplementary Table 1. Countries/Regions and number of papers published on tendon stem cells from 1991 to 2020.

Countries/Regions	Number of papers
USA	938
PEOPLES R CHINA	567
ENGLAND	219
GERMANY	195
ITALY	173
JAPAN	163
SOUTH KOREA	91
SWITZERLAND	82
AUSTRALIA	59
FRANCE	56
SINGAPORE	53
SPAIN	52
CANADA	50
PORTUGAL	46
BRAZIL	43
NETHERLANDS	37
TAIWAN	34
AUSTRIA	32
IRAN	26
TURKEY	26
IRELAND	25
SWEDEN	25
POLAND	24
INDIA	22
SCOTLAND	22
ISRAEL	21
BELGIUM	14
DENMARK	12
MALAYSIA	12
BULGARIA	10
GREECE	8
CHILE	6
NORWAY	5
INDONESIA	4
MEXICO	4
SAUDI ARABIA	4
COLOMBIA	3
FINLAND	3
IRAQ	3
NEW ZEALAND	3
NORTH IRELAND	3

RUSSIA	3
WALES	3
CZECH REPUBLIC	2
EGYPT	2
HUNGARY	2
SERBIA	2
ARGENTINA	1
CROATIA	1
CYPRUS	1
ETHIOPIA	1
GRENADA	1
JORDAN	1
LEBANON	1
LITHUANIA	1
NIGERIA	1
PAKISTAN	1
PALESTINE	1
ROMANIA	1
SLOVAKIA	1
SLOVENIA	1
SOUTH AFRICA	1
THAILAND	1
TRINIDAD TOBAGO	1
URUGUAY	1
