# Supplementary material for: Global Research Trends in Tendon Stem Cells from 1991 to 2020: A Bibliometric and Visualized Study
Source: Stem Cells Int. 2022 Jun 18;2022:7937765. doi: 10.1155/2022/7937765 (PMC9233735; doi:10.1155/2022/7937765)
Supplement: Supplementary 2 — Supplementary Table 2: journals and number of papers published on tendon stem cells from 1991 to 2020. [file 7937765.f2.pdf]

Supplementary Table 2. Journals and number of papers published on tendon stem cells from 1991 to 2020.

Journals	Number of papers
JOURNAL OF ORTHOPAEDIC RESEARCH	100
AMERICAN JOURNAL OF SPORTS MEDICINE	83
TISSUE ENGINEERING PART A	77
BIOMATERIALS	63
ACTA BIOMATERIALIA	51
PLOS ONE	49
STEM CELLS INTERNATIONAL	49
STEM CELL RESEARCH THERAPY	46
JOURNAL OF TISSUE ENGINEERING AND REGENERATIVE MEDI(	38
ARTHROSCOPY THE JOURNAL OF ARTHROSCOPIC AND RELATE	37
KNEE SURGERY SPORTS TRAUMATOLOGY ARTHROSCOPY	36
SCIENTIFIC REPORTS	33
CURRENT STEM CELL RESEARCH THERAPY	30
INTERNATIONAL JOURNAL OF MOLECULAR SCIENCES	30
CONNECTIVE TISSUE RESEARCH	28
CELL TRANSPLANTATION	27
JOURNAL OF SHOULDER AND ELBOW SURGERY	27
JOURNAL OF BIOMEDICAL MATERIALS RESEARCH PART A	26
TISSUE ENGINEERING	24
STEM CELLS AND DEVELOPMENT	23
EQUINE VETERINARY JOURNAL	20
BIOCHEMICAL AND BIOPHYSICAL RESEARCH COMMUNICATIOI	19
JOURNAL OF BONE AND JOINT SURGERY AMERICAN VOLUME	19
TISSUE ENGINEERING PART B REVIEWS	19
CLINICAL ORTHOPAEDICS AND RELATED RESEARCH	18
PLASTIC AND RECONSTRUCTIVE SURGERY	18
JOURNAL OF HAND SURGERY AMERICAN VOLUME	17
BMC MUSCULOSKELETAL DISORDERS	16
JOURNAL OF CELLULAR PHYSIOLOGY	16
REGENERATIVE MEDICINE	16
INTERNATIONAL ORTHOPAEDICS	15
JOURNAL OF ORTHOPAEDIC SURGERY AND RESEARCH	15
STEM CELLS	15
TISSUE ENGINEERING PART C METHODS	15
ADVANCES IN EXPERIMENTAL MEDICINE AND BIOLOGY	14
CELLS	14
JOURNAL OF BIOMEDICAL MATERIALS RESEARCH PART B APPI	14
ADVANCED HEALTHCARE MATERIALS	13
BIOMED RESEARCH INTERNATIONAL	13
BIOMEDICAL MATERIALS	13
CELLULAR PHYSIOLOGY AND BIOCHEMISTRY	13
CYTOTHERAPY	13

TISSUE ENGINEERING AND REGENERATIVE MEDICINE	13
BONE JOINT RESEARCH	12
FRONTIERS IN VETERINARY SCIENCE	12
STEM CELLS TRANSLATIONAL MEDICINE	12
CELL AND TISSUE RESEARCH	11
EUROPEAN CELLS MATERIALS	11
JOURNAL OF CELLULAR AND MOLECULAR MEDICINE	11
JOURNAL OF EQUINE VETERINARY SCIENCE	11
JOURNAL OF THE MECHANICAL BEHAVIOR OF BIOMEDICAL MA	11
MATERIALS SCIENCE ENGINEERING C MATERIALS FOR BIOLOG	11
STEM CELL REVIEWS AND REPORTS	11
ANNALS OF BIOMEDICAL ENGINEERING	10
ANNALS OF THE NEW YORK ACADEMY OF SCIENCES	10
JOURNAL OF BIOMECHANICS	10
JOURNAL OF MATERIALS CHEMISTRY B	10
JOURNAL OF THE AMERICAN ACADEMY OF ORTHOPAEDIC SUR	10
ACS BIOMATERIALS SCIENCE ENGINEERING	9
AMERICAN JOURNAL OF VETERINARY RESEARCH	9
EXPERT OPINION ON BIOLOGICAL THERAPY	9
OPERATIVE TECHNIQUES IN SPORTS MEDICINE	9
DEVELOPMENT	8
FASEB JOURNAL	8
FRONTIERS IN BIOENGINEERING AND BIOTECHNOLOGY	8
JOURNAL OF BONE AND MINERAL RESEARCH	8
JOURNAL OF MATERIALS SCIENCE MATERIALS IN MEDICINE	8
JOURNAL OF ORTHOPAEDIC TRANSLATION	8
MATERIALS	8
MOLECULAR MEDICINE REPORTS	8
RESEARCH IN VETERINARY SCIENCE	8
ACS APPLIED MATERIALS INTERFACES	7
ADVANCED DRUG DELIVERY REVIEWS	7
BIOFABRICATION	7
BIOMATERIALS SCIENCE	7
BONE	7
CLINICS IN SPORTS MEDICINE	7
EUROPEAN REVIEW FOR MEDICAL AND PHARMACOLOGICAL S	7
HISTOLOGY AND HISTOPATHOLOGY	7
INTERNATIONAL JOURNAL OF NANOMEDICINE	7
JOURNAL OF BIOMATERIALS SCIENCE POLYMER EDITION	7
JOURNAL OF CELLULAR BIOCHEMISTRY	7
JOURNAL OF TISSUE ENGINEERING	7
JOVE JOURNAL OF VISUALIZED EXPERIMENTS	7
MEDICAL SCIENCE MONITOR	7
SPORTS MEDICINE AND ARTHROSCOPY REVIEW	7
VETERINARY CLINICS OF NORTH AMERICA EQUINE PRACTICE	7

VETERINARY JOURNAL	7
BRITISH MEDICAL BULLETIN	6
CELLS TISSUES ORGANS	6
DEVELOPMENTAL BIOLOGY	6
EXPERIMENTAL CELL RESEARCH	6
FRONTIERS IN CELL AND DEVELOPMENTAL BIOLOGY	6
JOURNAL OF BIOMATERIALS AND TISSUE ENGINEERING	6
JOURNAL OF BIOMECHANICAL ENGINEERING TRANSACTIONS (	6
MATRIX BIOLOGY	6
PM R	6
PROCEEDINGS OF THE NATIONAL ACADEMY OF SCIENCES OF T	6
VETERINARY AND COMPARATIVE ORTHOPAEDICS AND TRAUM	6
VETERINARY RESEARCH COMMUNICATIONS	6
VETERINARY SURGERY	6
BIOTECHNOLOGY AND BIOENGINEERING	5
BMC CELL BIOLOGY	5
CELL BIOLOGY INTERNATIONAL	5
CELL PROLIFERATION	5
CHINESE MEDICAL JOURNAL	5
CYTOTECHNOLOGY	5
FRONTIERS IN BIOSCIENCE LANDMARK	5
JOURNAL OF ANATOMY	5
JOURNAL OF APPLIED PHYSIOLOGY	5
JOURNAL OF CLINICAL INVESTIGATION	5
METABOLIC INFLUENCES ON RISK FOR TENDON DISORDERS	5
ONCOTARGET	5
ORTHOPAEDIC JOURNAL OF SPORTS MEDICINE	5
ADVANCED FUNCTIONAL MATERIALS	4
AMERICAN JOURNAL OF PHYSIOLOGY CELL PHYSIOLOGY	4
ANNUAL REVIEW OF BIOMEDICAL ENGINEERING	4
ARCHIVOS DE MEDICINA VETERINARIA	4
BIOLOGY OPEN	4
BIOTECHNOLOGY LETTERS	4
BIRTH DEFECTS RESEARCH PART C EMBRYO TODAY REVIEWS	4
BMC VETERINARY RESEARCH	4
BONE RESEARCH	4
CURRENT GENE THERAPY	4
CURRENT PHARMACEUTICAL DESIGN	4
CYTOMETRY PART A	4
DIFFERENTIATION	4
EXPERIMENTAL AND THERAPEUTIC MEDICINE	4
FRONTIERS IN PHYSIOLOGY	4
IN VITRO CELLULAR DEVELOPMENTAL BIOLOGY ANIMAL	4
INJURY INTERNATIONAL JOURNAL OF THE CARE OF THE INJUR	4
INTERNATIONAL JOURNAL OF CLINICAL AND EXPERIMENTAL I	4

INTERNATIONAL JOURNAL OF MOLECULAR MEDICINE	4
JOURNAL OF BIOLOGICAL CHEMISTRY	4
JOURNAL OF MUSCULOSKELETAL NEURONAL INTERACTIONS	4
JOURNAL OF NANOMATERIALS	4
JOURNAL OF ORTHOPAEDIC SCIENCE	4
JOURNAL OF TRANSLATIONAL MEDICINE	4
MACROMOLECULAR BIOSCIENCE	4
MUSCULOSKELETAL REPAIR AND REGENERATION	4
NATURE REVIEWS RHEUMATOLOGY	4
PFERDEHEILKUNDE	4
PHYSICAL MEDICINE AND REHABILITATION CLINICS OF NORTI	4
POLYMERS	4
REGENERATIVE THERAPY	4
TRENDS IN BIOTECHNOLOGY	4
WORLD JOURNAL OF STEM CELLS	4
ACTA OF BIOENGINEERING AND BIOMECHANICS	3
ADVANCED MATERIALS	3
AGING CELL	3
ANNALS OF PLASTIC SURGERY	3
ARCHIVES OF ORTHOPAEDIC AND TRAUMA SURGERY	3
ARTHRITIS RESEARCH THERAPY	3
ARTIFICIAL ORGANS	3
BIOACTIVE MATERIALS	3
BIOTECHNOLOGY AND BIOPROCESS ENGINEERING	3
BIOTECHNOLOGY JOURNAL	3
BRITISH JOURNAL OF SPORTS MEDICINE	3
CALCIFIED TISSUE INTERNATIONAL	3
CANADIAN VETERINARY JOURNAL REVUE VETERINAIRE CANA	3
CLINICS IN PODIATRIC MEDICINE AND SURGERY	3
CURRENT TOPICS IN DEVELOPMENTAL BIOLOGY	3
DEVELOPMENTAL DYNAMICS	3
DISABILITY AND REHABILITATION	3
ELIFE	3
EXPERIMENTAL NEUROLOGY	3
FOOT AND ANKLE CLINICS	3
INTERNATIONAL JOURNAL OF ARTIFICIAL ORGANS	3
INTERNATIONAL JOURNAL OF EXPERIMENTAL PATHOLOGY	3
INTERNATIONAL JOURNAL OF STEM CELLS	3
JCI INSIGHT	3
JOURNAL OF BIOMATERIALS APPLICATIONS	3
JOURNAL OF BONE AND JOINT SURGERY BRITISH VOLUME	3
JOURNAL OF HAND SURGERY EUROPEAN VOLUME	3
JOURNAL OF PLASTIC RECONSTRUCTIVE AND AESTHETIC SUR(	3
LASERS IN MEDICAL SCIENCE	3
MEDICAL HYPOTHESES	3

MOLECULAR AND CELLULAR BIOCHEMISTRY	3
MOLECULAR BIOLOGY REPORTS	3
NANOMEDICINE	3
NANOMEDICINE NANOTECHNOLOGY BIOLOGY AND MEDICINE	3
NATURE COMMUNICATIONS	3
NATURE MEDICINE	3
ORTHOPEDIC CLINICS OF NORTH AMERICA	3
PLATELETS	3
POLISH JOURNAL OF VETERINARY SCIENCES	3
RHEUMATOLOGY	3
SCANDINAVIAN JOURNAL OF MEDICINE SCIENCE IN SPORTS	3
SCIENCE ADVANCES	3
VETERINARY QUARTERLY	3
WILEY INTERDISCIPLINARY REVIEWS DEVELOPMENTAL BIOLC	3
ACTA BIOCHIMICA ET BIOPHYSICA SINICA	2
ACTA CIRURGICA BRASILEIRA	2
ACTA ORTHOPAEDICA ET TRAUMATOLOGICA TURCICA	2
ADVANCED SCIENCE	2
ADVANCED THERAPEUTICS	2
ADVANCES IN BIOCHEMICAL ENGINEERING BIOTECHNOLOGY	2
AGING US	2
AMERICAN JOURNAL OF PATHOLOGY	2
AMERICAN JOURNAL OF PHYSICAL MEDICINE REHABILITATIOI	2
AMERICAN JOURNAL OF TRANSLATIONAL RESEARCH	2
ANNALS OF TRANSLATIONAL MEDICINE	2
ARCHIVES OF MEDICAL SCIENCE	2
BIO MEDICAL MATERIALS AND ENGINEERING	2
BIOMACROMOLECULES	2
BIOMECHANICS AND MODELING IN MECHANOBIOLOGY	2
BIOMEDICAL ENGINEERING ONLINE	2
BIOTECHNOLOGY ADVANCES	2
BONE JOINT JOURNAL	2
CANADIAN JOURNAL OF VETERINARY RESEARCH REVUE CANA	2
CELL AND TISSUE BANKING	2
CELL BIOCHEMISTRY AND BIOPHYSICS	2
CELL CYCLE	2
CELL DEATH DISEASE	2
CELL JOURNAL	2
CLINICAL JOURNAL OF SPORT MEDICINE	2
CLINICS IN PLASTIC SURGERY	2
COLLOIDS AND SURFACES B BIOINTERFACES	2
CURRENT PHARMACEUTICAL BIOTECHNOLOGY	2
DENTAL MATERIALS	2
DEVELOPMENT GROWTH DIFFERENTIATION	2
DISCOVERY MEDICINE	2

ENGINEERING MINERALIZED AND LOAD BEARING TISSUES	2
EQUINE VETERINARY EDUCATION	2
EXPERIMENTAL BIOLOGY AND MEDICINE	2
FOLIA MORPHOLOGICA	2
FRONTIERS IN AGING NEUROSCIENCE	2
FRONTIERS IN PHARMACOLOGY	2
GENE	2
GROWTH FACTORS	2
IN VIVO	2
INDIAN JOURNAL OF ANIMAL SCIENCES	2
INTERNATIONAL JOURNAL OF BIOCHEMISTRY CELL BIOLOGY	2
INTERNATIONAL JOURNAL OF BIOLOGICAL MACROMOLECULE	2
INTERNATIONAL JOURNAL OF MORPHOLOGY	2
INTERNATIONAL JOURNAL OF ORAL SCIENCE	2
IRANIAN JOURNAL OF BASIC MEDICAL SCIENCES	2
JOINT BONE SPINE	2
JOURNAL OF APPLIED BIOMATERIALS FUNCTIONAL MATERIAL	2
JOURNAL OF BIOMEDICAL MATERIALS RESEARCH	2
JOURNAL OF BIOMEDICAL NANOTECHNOLOGY	2
JOURNAL OF BIOMEDICAL SCIENCE	2
JOURNAL OF BONE AND MINERAL METABOLISM	2
JOURNAL OF CELL BIOLOGY	2
JOURNAL OF CELL SCIENCE	2
JOURNAL OF CLINICAL MEDICINE	2
JOURNAL OF GENE MEDICINE	2
JOURNAL OF MOLECULAR HISTOLOGY	2
JOURNAL OF NUTRITION HEALTH AGING	2
JOURNAL OF ORTHOPAEDIC SURGERY	2
JOURNAL OF PAIN RESEARCH	2
JOURNAL OF PEDIATRIC SURGERY	2
JOURNAL OF STEROID BIOCHEMISTRY AND MOLECULAR BIOL(	2
JOURNAL OF SURGICAL RESEARCH	2
JOURNAL OF THE HELLENIC VETERINARY MEDICAL SOCIETY	2
JOURNAL OF THE ROYAL SOCIETY INTERFACE	2
KNEE	2
LABORATORY INVESTIGATION	2
MATERIALS SCIENCE ENGINEERING C BIOMIMETIC AND SUPRA	2
MEDICAL ENGINEERING PHYSICS	2
MEDICINE	2
MICROSCOPY RESEARCH AND TECHNIQUE	2
MOLECULAR PHARMACEUTICS	2
MOLECULAR THERAPY	2
NANOSCALE	2
NEUROSCIENCE	2
NPJ REGENERATIVE MEDICINE	2

ORTHOPAEDICS TRAUMATOLOGY SURGERY RESEARCH	2
ORTHOPEDICS	2
OXIDATIVE MEDICINE AND CELLULAR LONGEVITY	2
PEERJ	2
PHARMACEUTICS	2
PHYSICIAN AND SPORTSMEDICINE	2
PROCEEDINGS OF THE INSTITUTION OF MECHANICAL ENGINEE	2
PROTEOMICS	2
PROTEOMICS CLINICAL APPLICATIONS	2
REJUVENATION RESEARCH	2
RSC ADVANCES	2
SCIENTIST	2
SEMINARS IN ARTHRITIS AND RHEUMATISM	2
SPORTS MEDICINE	2
STEM CELL RESEARCH	2
TECHNOLOGY AND HEALTH CARE	2
THERIOGENOLOGY	2
TOXICOLOGY RESEARCH	2
TRANSLATIONAL RESEARCH	2
TRANSPLANTATION	2
TRANSPLANTATION PROCEEDINGS	2
VERTEBRATE SKELETAL DEVELOPMENT	2
VETERINARY MEDICINE AND SCIENCE	2
WOUND REPAIR AND REGENERATION	2
ACS CHEMICAL BIOLOGY	1
ACTA PHYSIOLOGICA	1
ACTA VETERINARIA BRNO	1
ADVANCES IN CLINICAL AND EXPERIMENTAL MEDICINE	1
ADVANCES IN POLYMER SCIENCE	1
AESTHETIC SURGERY JOURNAL	1
AMERICAN JOURNAL OF MEDICAL GENETICS	1
AMERICAN JOURNAL OF ROENTGENOLOGY	1
AMERICAN JOURNAL OF SURGICAL PATHOLOGY	1
ANATOMY AND EMBRYOLOGY	1
ANKARA UNIVERSITESI VETERINER FAKULTESI DERGISI	1
ANNALS ACADEMY OF MEDICINE SINGAPORE	1
ANNALS OF HEMATOLOGY	1
ANNALS OF THE RHEUMATIC DISEASES	1
ANNUAL REVIEW OF BIOMEDICAL ENGINEERING VOL 14	1
ANNUAL REVIEW OF BIOMEDICAL ENGINEERING VOL 15	1
ANNUAL REVIEW OF BIOMEDICAL ENGINEERING VOL 20	1
ANNUAL REVIEW OF CELL AND DEVELOPMENTAL BIOLOGY	1
ANTI CANCER DRUGS	1
APPLIED BIONICS AND BIOMECHANICS	1
APPLIED MATERIALS TODAY	1

APPLIED SCIENCES BASEL	1
ARCHIVES OF ORAL BIOLOGY	1
ARCHIVES OF PATHOLOGY LABORATORY MEDICINE	1
ARCHIVES OF PHARMACAL RESEARCH	1
ARCHIVUM IMMUNOLOGIAE ET THERAPIAE EXPERIMENTALIS	1
ARQUIVO BRASILEIRO DE MEDICINA VETERINARIA E ZOOTECN	1
ARTHRITIS AND RHEUMATISM	1
AUSTRALIAN DENTAL JOURNAL	1
BEST PRACTICE RESEARCH IN CLINICAL RHEUMATOLOGY	1
BIOCHEMICAL ENGINEERING JOURNAL	1
BIOCHEMICAL PHARMACOLOGY	1
BIOCONJUGATE CHEMISTRY	1
BIOENGINEERING BASEL	1
BIOLOGICAL PHARMACEUTICAL BULLETIN	1
BIOMEDICAL APPLICATIONS OF POLYMERIC NANOFIBERS	1
BIOMEDICAL MICRODEVICES	1
BIOMEDICAL OPTICS EXPRESS	1
BIOMEDICINE PHARMACOTHERAPY	1
BIOMEDICINES	1
BIOMOLECULES	1
BIOPHYSICAL JOURNAL	1
BIOREACTOR SYSTEMS FOR TISSUE ENGINEERING	1
BIOREACTOR SYSTEMS FOR TISSUE ENGINEERING II STRATEGI	1
BIOSCIENCE REPORTS	1
BIOTECHNOLOGY AND APPLIED BIOCHEMISTRY	1
BIOTECHNOLOGY PROGRESS	1
BMC BIOTECHNOLOGY	1
BMC COMPLEMENTARY AND ALTERNATIVE MEDICINE	1
BMC DEVELOPMENTAL BIOLOGY	1
BMC MOLECULAR BIOLOGY	1
BMJ OPEN	1
BONE MORPHOGENIC PROTEIN	1
BRAZILIAN JOURNAL OF MEDICAL AND BIOLOGICAL RESEARC	1
BREAST CANCER RESEARCH	1
BRITISH JOURNAL OF DERMATOLOGY	1
BRITISH JOURNAL OF HAEMATOLOGY	1
BRITISH JOURNAL OF HOSPITAL MEDICINE	1
BRITISH JOURNAL OF PHARMACOLOGY	1
BRITISH JOURNAL OF RADIOLOGY	1
BULLETIN OF EXPERIMENTAL BIOLOGY AND MEDICINE	1
CANADIAN JOURNAL OF PHYSIOLOGY AND PHARMACOLOGY	1
CANCER CELL	1
CARDIOVASCULAR DRUGS AND THERAPY	1
CELL	1
CELL BIOLOGY AND TRANSLATIONAL MEDICINE VOL 2 APPRO	1

CELL BIOLOGY AND TRANSLATIONAL MEDICINE VOL 5 STEM C	1
CELLULAR AND MOLECULAR BIOENGINEERING	1
CELLULAR AND MOLECULAR BIOLOGY	1
CELLULAR AND MOLECULAR LIFE SCIENCES	1
CHEMICAL ENGINEERING JOURNAL	1
CHEMICAL REVIEWS	1
CHEMISTRY AND PHYSICS OF LIPIDS	1
CHINESE JOURNAL OF PHYSIOLOGY	1
CLINICA TERAPEUTICA	1
CLINICAL ANATOMY	1
CLINICAL AND EXPERIMENTAL PHARMACOLOGY AND PHYSIOI	1
CLINICAL AND EXPERIMENTAL RHEUMATOLOGY	1
CLINICAL BIOMECHANICS	1
CLINICAL CANCER RESEARCH	1
CLINICAL SCIENCE	1
CLINICS IN DERMATOLOGY	1
COLD SPRING HARBOR PERSPECTIVES IN BIOLOGY	1
COMPARATIVE MEDICINE	1
COMPTES RENDUS DE L ACADEMIE BULGARE DES SCIENCES	1
CONTRAST MEDIA MOLECULAR IMAGING	1
CRITICAL REVIEWS IN THERAPEUTIC DRUG CARRIER SYSTEMS	1
CURRENT MEDICINAL CHEMISTRY	1
CURRENT OPINION IN BIOTECHNOLOGY	1
CURRENT OPINION IN IMMUNOLOGY	1
CURRENT ORTHOPAEDICS	1
CURRENT RHEUMATOLOGY REPORTS	1
CYTOKINE GROWTH FACTOR REVIEWS	1
DERMATOLOGY	1
DEUTSCHE ZEITSCHRIFT FUR SPORTMEDIZIN	1
DIABETES METABOLISM RESEARCH AND REVIEWS	1
DISEASE MODELS MECHANISMS	1
EBIOMEDICINE	1
EFORT OPEN REVIEWS	1
EUROPEAN BIOPHYSICS JOURNAL WITH BIOPHYSICS LETTERS	1
EUROPEAN JOURNAL OF HISTOCHEMISTRY	1
EXPERIMENTAL AND MOLECULAR MEDICINE	1
EXPERIMENTAL EYE RESEARCH	1
EXPERIMENTAL HEMATOLOGY	1
EXPERIMENTAL MECHANICS	1
EXPERT OPINION ON DRUG SAFETY	1
EXPERT OPINION ON ORPHAN DRUGS	1
EXPERT OPINION ON PHARMACOTHERAPY	1
EXPERT REVIEW OF MEDICAL DEVICES	1
FEBS LETTERS	1
FEBS OPEN BIO	1

FOOT AND ANKLE SURGERY	1
FOOT ANKLE INTERNATIONAL	1
FRONTIERS IN CHEMISTRY	1
FRONTIERS IN GENETICS	1
FRONTIERS IN INTEGRATIVE NEUROSCIENCE	1
GENE THERAPY	1
GENES	1
GENESIS	1
GENETICS AND MOLECULAR RESEARCH	1
GROWTH HORMONE IGF RESEARCH	1
HAND CLINICS	1
HIP INTERNATIONAL	1
HISTOCHEMISTRY AND CELL BIOLOGY	1
HUMAN CELL	1
HUMAN GENE THERAPY	1
IEEE TRANSACTIONS ON NANOBIOSCIENCE	1
ILAR JOURNAL	1
INDIAN JOURNAL OF ORTHOPAEDICS	1
INFLAMMATION	1
INTERNATIONAL IMMUNOPHARMACOLOGY	1
INTERNATIONAL JOURNAL OF APPLIED RESEARCH IN VETERIN	1
INTERNATIONAL JOURNAL OF DEVELOPMENTAL BIOLOGY	1
INTERNATIONAL JOURNAL OF HUMAN GENETICS	1
INTERNATIONAL JOURNAL OF IMMUNOPATHOLOGY AND PHAI	1
INTERNATIONAL JOURNAL OF MEDICAL SCIENCES	1
INTERNATIONAL JOURNAL OF ORAL AND MAXILLOFACIAL SUI	1
INTERNATIONAL JOURNAL OF PEPTIDE RESEARCH AND THERA	1
INTERNATIONAL JOURNAL OF PHARMACEUTICS	1
INTERNATIONAL JOURNAL OF SHOULDER SURGERY	1
INTERNATIONAL JOURNAL OF SPORTS MEDICINE	1
INTERNATIONAL REVIEW OF CELL AND MOLECULAR BIOLOGY	1
INTERNATIONAL REVIEW OF CELL AND MOLECULAR BIOLOGY	1
IRANIAN JOURNAL OF VETERINARY RESEARCH	1
ISRAEL MEDICAL ASSOCIATION JOURNAL	1
IUBMB LIFE	1
JOURNAL OF ANIMAL AND VETERINARY ADVANCES	1
JOURNAL OF ANIMAL SCIENCE	1
JOURNAL OF BIOLOGICAL ENGINEERING	1
JOURNAL OF BIOLOGICAL REGULATORS AND HOMEOSTATIC A	1
JOURNAL OF BIOLOGICAL RESEARCH THESSALONIKI	1
JOURNAL OF BIOMEDICAL OPTICS	1
JOURNAL OF BIOSCIENCE AND BIOENGINEERING	1
JOURNAL OF CLINICAL PERIODONTOLOGY	1
JOURNAL OF COMPARATIVE PATHOLOGY	1
JOURNAL OF CRANIO MAXILLOFACIAL SURGERY	1

JOURNAL OF CUTANEOUS PATHOLOGY	1
JOURNAL OF DENTAL RESEARCH	1
JOURNAL OF ENVIRONMENTAL PROTECTION AND ECOLOGY	1
JOURNAL OF EXPERIMENTAL BIOLOGY	1
JOURNAL OF FORENSIC SCIENCES	1
JOURNAL OF HAND THERAPY	1
JOURNAL OF HEMATOTHERAPY STEM CELL RESEARCH	1
JOURNAL OF HISTOCHEMISTRY CYTOCHEMISTRY	1
JOURNAL OF HUAZHONG UNIVERSITY OF SCIENCE AND TECHN	1
JOURNAL OF IMMUNOLOGY	1
JOURNAL OF INDUSTRIAL AND ENGINEERING CHEMISTRY	1
JOURNAL OF INFLAMMATION RESEARCH	1
JOURNAL OF INTERNATIONAL MEDICAL RESEARCH	1
JOURNAL OF MATERIALS SCIENCE	1
JOURNAL OF MECHANICS IN MEDICINE AND BIOLOGY	1
JOURNAL OF MEDICAL IMAGING AND HEALTH INFORMATICS	1
JOURNAL OF MEDICINAL FOOD	1
JOURNAL OF MOLECULAR CELL BIOLOGY	1
JOURNAL OF MORPHOLOGY	1
JOURNAL OF MUSCLE RESEARCH AND CELL MOTILITY	1
JOURNAL OF NANOBIOTECHNOLOGY	1
JOURNAL OF NANOSCIENCE AND NANOTECHNOLOGY	1
JOURNAL OF NEUROPHYSIOLOGY	1
JOURNAL OF PATHOLOGY	1
JOURNAL OF PERIODONTAL RESEARCH	1
JOURNAL OF PHOTOCHEMISTRY AND PHOTOBIOLOGY B BIOLO	1
JOURNAL OF PLASTIC SURGERY AND HAND SURGERY	1
JOURNAL OF PROTEOME RESEARCH	1
JOURNAL OF SPORTS MEDICINE AND PHYSICAL FITNESS	1
JOURNAL OF THE NEUROLOGICAL SCIENCES	1
JOURNAL OF VETERINARY MEDICAL SCIENCE	1
JOURNAL OF VETERINARY SCIENCE	1
JOURNAL OF WOUND CARE	1
JOURNAL OF ZHEJIANG UNIVERSITY SCIENCE B	1
KAFKAS UNIVERSITESI VETERINER FAKULTESI DERGISI	1
LABORATORY ANIMALS	1
LANCET HAEMATOLOGY	1
LIFE SCIENCE JOURNAL ACTA ZHENGZHOU UNIVERSITY OVER	1
LIFE SCIENCES	1
MACROMOLECULAR RAPID COMMUNICATIONS	1
MARINE DRUGS	1
MATERIALWISSENSCHAFT UND WERKSTOFFTECHNIK	1
MEASUREMENT SCIENCE AND TECHNOLOGY	1
MECHANISMS OF AGEING AND DEVELOPMENT	1
MEDICINA LITHUANIA	1

METHODS	1
METHODS IN CELL BIOLOGY	1
MICRO NANO LETTERS	1
MICROMACHINES	1
MICRON	1
MICROSYSTEMS NANOENGINEERING	1
MODERN RHEUMATOLOGY	1
MOLECULAR AND CELLULAR BIOLOGY	1
MOLECULAR BIOTECHNOLOGY	1
MOLECULAR CELL	1
MOLECULAR PAIN	1
MOLECULES	1
MRS COMMUNICATIONS	1
NANO LETTERS	1
NANOMATERIALS	1
NANOSCALE ADVANCES	1
NATURE CELL BIOLOGY	1
NATURE PROTOCOLS	1
NATURWISSENSCHAFTEN	1
NEUROPATHOLOGY	1
NEUROSURGICAL FOCUS	1
NEW ZEALAND VETERINARY JOURNAL	1
NMR IN BIOMEDICINE	1
ONCOLOGY LETTERS	1
OPEN LIFE SCIENCES	1
ORAL DISEASES	1
ORGAN DEVELOPMENT	1
ORGANOGENESIS	1
OSTEOARTHRITIS AND CARTILAGE	1
OSTEOCHONDRAL TISSUE ENGINEERING NANOTECHNOLOGY S	1
PAIN AND THERAPY	1
PAIN MEDICINE	1
PAIN PHYSICIAN	1
PAKISTAN VETERINARY JOURNAL	1
PATHOBIOLOGY	1
PATHOLOGY	1
PATHOLOGY RESEARCH AND PRACTICE	1
PEDIATRIC HEMATOLOGY AND ONCOLOGY	1
PEDIATRIC RESEARCH	1
PESQUISA VETERINARIA BRASILEIRA	1
PHARMACEUTICAL BIOLOGY	1
PHOTOCHEMISTRY AND PHOTOBIOLOGY	1
PHYSICAL THERAPY	1
PLOS NEGLECTED TROPICAL DISEASES	1
POLYMER TESTING	1

POLYMERS FOR ADVANCED TECHNOLOGIES	1
PREPARATIVE BIOCHEMISTRY BIOTECHNOLOGY	1
PROGRESS IN HERITABLE SOFT CONNECTIVE TISSUE DISEASES	1
PROGRESS IN NATURAL SCIENCE MATERIALS INTERNATIONAL	1
PROGRESS IN POLYMER SCIENCE	1
RADIOLOGY	1
RAPID PROTOTYPING JOURNAL	1
REPRODUCTION IN DOMESTIC ANIMALS	1
REVIEW OF SCIENTIFIC INSTRUMENTS	1
RHEUMATOLOGY INTERNATIONAL	1
SAINS MALAYSIANA	1
SCANDINAVIAN JOURNAL OF PLASTIC AND RECONSTRUCTIVE	1
SCIENCE	1
SEMINA CIENCIAS AGRARIAS	1
SEMINARS IN PEDIATRIC SURGERY	1
SEMINARS IN PLASTIC SURGERY	1
SEMINARS IN THROMBOSIS AND HEMOSTASIS	1
SENSORS AND ACTUATORS B CHEMICAL	1
SKELETAL BIOLOGY AND MEDICINE PT B	1
SLAS TECHNOLOGY	1
SLOVENIAN VETERINARY RESEARCH	1
SMALL	1
SPINE	1
STEM CELL REVIEWS	1
SURGEON JOURNAL OF THE ROYAL COLLEGES OF SURGEONS C	1
SURGICAL AND RADIOLOGIC ANATOMY	1
SURGICAL INNOVATION	1
SWISS MEDICAL WEEKLY	1
THERANOSTICS	1
TISSUE CELL	1
TRANSFUSION AND APHERESIS SCIENCE	1
TRENDS IN CARDIOVASCULAR MEDICINE	1
TRENDS IN MOLECULAR MEDICINE	1
TRIALS	1
ULTRASCHALL IN DER MEDIZIN	1
ULTRASONICS	1
ULTRASOUND QUARTERLY	1
ULUSAL TRAVMA VE ACIL CERRAHI DERGISI TURKISH JOURNA	1
VETERINARY CLINICAL PATHOLOGY	1
VETERINARY IMMUNOLOGY AND IMMUNOPATHOLOGY	1
VETERINARY RADIOLOGY ULTRASOUND	1
VETERINARY RESEARCH FORUM	1
VITAMINS AND HORMONES	1
VLAAMS DIERGENEESKUNDIG TIJDSCHRIFT	1
VOJNOSANITETSKI PREGLED	1

WORLD JOURNAL OF GASTROENTEROLOGY	1
WORLD NEUROSURGERY	1
ZEBRAFISH DISEASE MODELS AND CHEMICAL SCREENS	1
ZEITSCHRIFT FUR ORTHOPADIE UND IHRE GRENZGEBIETE	1
