# Supplementary material for: Global Research Trends in Tendon Stem Cells from 1991 to 2020: A Bibliometric and Visualized Study
Source: Stem Cells Int. 2022 Jun 18;2022:7937765. doi: 10.1155/2022/7937765 (PMC9233735; doi:10.1155/2022/7937765)
Supplement: Supplementary 3 — Supplementary Table 3: institutions and number of papers published on tendon stem cells from 1991 to 2020. [file 7937765.f3.pdf]

Supplementary Table 3. Institutions and number of papers published on tendon stem cells from 1991 to 2020.

Institutions	Number of papers
UNIV PITTSBURGH	96
ZHEJIANG UNIV	70
CHINESE UNIV HONG KONG	64
SHANGHAI JIAO TONG UNIV	64
STANFORD UNIV	50
MAYO CLIN	48
UNIV CONNECTICUT	45
NATL UNIV SINGAPORE	44
HOSP SPECIAL SURG	40
UNIV MINHO	39
HARVARD UNIV	38
THIRD MIL MED UNIV	37
UNIV CINCINNATI	36
UNIV PENN	36
COLUMBIA UNIV	35
UNIV ILLINOIS	33
UNIV MILAN	31
CORNELL UNIV	30
WASHINGTON UNIV	30
QUEEN MARY UNIV LONDON	27
ICVS 3BS PT GOVT ASSOCIATE LAB	25
CASE WESTERN RESERVE UNIV	24
TONGJI UNIV	23
UNIV OXFORD	23
KEELE UNIV	22
SWISS FED INST TECHNOL	22
UNIV CALIF DAVIS	22
PEKING UNIV	21
SICHUAN UNIV	21
TECH UNIV MUNICH	21
CHINESE ACAD SCI	20
IRCCS IST ORTOPED GALEAZZI	20
SOUTHEAST UNIV	20
UNIV LIVERPOOL	20
UNIV MICHIGAN	20
UNIV REGENSBURG	20
COLORADO STATE UNIV	19
SOUTHERN MED UNIV	19
UNIV LEIPZIG	19
UNIV SALERNO	19

CHONGQING UNIV	18
HARVARD MED SCH	18
KOBE UNIV	18
SEOUL NATL UNIV	18
UNIV CALIF LOS ANGELES	18
UNIV COLL LONDON	18
UNIV HONG KONG	18
GEORGIA INST TECHNOL	17
VIRGINIA TECH	17
HIROSHIMA UNIV	16
KYOTO UNIV	16
NAGOYA UNIV	16
TUFTS UNIV	16
CHINA ORTHOPED REGENERAT MED GRP CORMED	15
TOKYO MED DENT UNIV	15
UCL	15
UNIV CAMBRIDGE	15
UNIV EDINBURGH	15
UNIV MANCHESTER	15
UNIV MUNICH	15
UNIV TERAMO	15
ANIM HLTH TRUST	14
FUDAN UNIV	14
HANNOVER MED SCH	14
SOOCHOW UNIV	14
UNIV G DANNUNZIO	14
UNIV ROCHESTER	14
UNIV ZURICH	14
EMORY UNIV	13
HOSP AUTHOR	13
ICAHN SCH MED MT SINAI	13
ROYAL NATL ORTHOPAED HOSP	13
ROYAL VET COLL	13
UNIV LONDON ROYAL VET COLL	13
UNIV WISCONSIN	13
VET AFFAIRS PALO ALTO HLTH CARE SYST	13
CHARITE	12
DONGGUK UNIV	12
DONGHUA UNIV	12
NATL TAIWAN UNIV	12
PURDUE UNIV	12
SECOND MIL MED UNIV	12
UNIV FLORIDA	12
UNIV PADUA	12
UNIV VIRGINIA	12

UNIV WESTERN AUSTRALIA	12
WENZHOU MED UNIV	12
CENT S UNIV	11
CHONGQING MED UNIV	11
HARBIN MED UNIV	11
HEADQUARTERS UNIV MINHO	11
JOHNS HOPKINS UNIV	11
MIT	11
NATL TISSUE ENGN CTR CHINA	11
NORTH CAROLINA STATE UNIV	11
PARACELSUS MED UNIV	11
PRINCE WALES HOSP	11
UNIV BOLOGNA	11
UNIV CALIF SAN DIEGO	11
YONSEI UNIV	11
ZHEJIANG PROV KEY LAB TISSUE ENGN REGENERAT MED	11
CHINA ORTHOPED REGENERAT MED GRP	10
NANJING MED UNIV	10
SAO PAULO STATE UNIV	10
SCRIPPS RES INST	10
UNIV CALIF SAN FRANCISCO	10
UNIV UTRECHT	10
CNR	9
DUKE UNIV	9
OHIO STATE UNIV	9
OSIRIS THERAPEUT INC	9
SHRINERS HOSP CHILDREN	9
TSINGHUA UNIV	9
UNIV GHENT	9
UNIV LONDON	9
VA PALO ALTO HLTH CARE SYST	9
BRIGHAM WOMENS HOSP	8
CAPITAL MED UNIV	8
CHINA MED UNIV	8
CRUCES UNIV HOSP	8
ETH	8
FREE UNIV BERLIN	8
INSERM	8
KAROLINSKA INST	8
KEY LAB ORGAN INJURY AGING REGENERAT MED HUNAN	8
KONKUK UNIV	8
LMU	8
MONASH UNIV	8
NANYANG TECHNOL UNIV	8
NATL UNIV IRELAND GALWAY	8

QUEENSLAND UNIV TECHNOL	8
RIZZOLI ORTHOPED INST	8
RUSH UNIV	8
STEADMAN PHILIPPON RES INST	8
TOHOKU UNIV	8
UMEA UNIV	8
UNIV GIESSEN	8
UNIV MARYLAND	8
UNIV VET MED HANNOVER	8
UNIV WASHINGTON	8
WROCLAW UNIV ENVIRONM LIFE SCI	8
WUHAN UNIV	8
XI AN JIAO TONG UNIV	8
ALBERT EINSTEIN COLL MED	7
BOSTON UNIV	7
CENT SOUTH UNIV	7
CHANG GUNG UNIV	7
CHARITE UNIV MED BERLIN	7
CHILDRENS HOSP PHILADELPHIA	7
CHINESE PEOPLES LIBERAT ARMY GEN HOSP	7
FERDOWSI UNIV MASHHAD	7
FOURTH MIL MED UNIV	7
HEBREW UNIV JERUSALEM	7
IRCCS POLICLIN SAN DONATO	7
JUNTENDO UNIV	7
MASSACHUSETTS GEN HOSP	7
MED UNIV PLOVDIV	7
MEDSTAR UNION MEM HOSP	7
MILE END HOSP	7
NORTHWESTERN UNIV	7
OKAYAMA UNIV	7
OSAKA UNIV	7
PENN STATE UNIV	7
SHANGHAI JIAO TONG UNIV AFFILIATED PEOPLES HOSP 6	7
SUN YAT SEN UNIV	7
TECHNION ISRAEL INST TECHNOL	7
THOMAS JEFFERSON UNIV	7
TULANE UNIV	7
UNIV BERN	7
UNIV COPENHAGEN	7
UNIV DELAWARE	7
UNIV GUELPH	7
UNIV KANSAS	7
UNIV LEEDS	7
UNIV MALAYA	7

UNIV MED CTR UTRECHT	7
UNIV SHEFFIELD	7
UNIV SO CALIF	7
UNIV SOUTHERN CALIF	7
UNIV TORONTO	7
WRIGHT STATE UNIV	7
ANHUI MED UNIV	6
AUSTRIAN CLUSTER TISSUE REGENERAT	6
CAMPUS BIOMED UNIV	6
CENTENO SCHULTZ CLIN	6
CHUNG ANG UNIV	6
CINCINNATI SPORTSMED ORTHOPAED CTR INC	6
CLEVELAND CLIN	6
CNRS	6
EINDHOVEN UNIV TECHNOL	6
HOKKAIDO UNIV	6
HUAZHONG UNIV SCI TECHNOL	6
JILIN UNIV	6
KYOTO PREFECTURAL UNIV MED	6
KYUNG HEE UNIV	6
LOUISIANA STATE UNIV	6
LUDWIG MAXIMILIANS UNIV MUNCHEN	6
NANJING UNIV	6
NANTONG UNIV	6
NATL TAIWAN UNIV HOSP	6
NYU	6
OREGON HLTH SCI UNIV	6
RUTGERS STATE UNIV	6
SHANDONG UNIV	6
SHANGHAI SIXTH PEOPLES HOSP	6
STEADMAN CLIN	6
SUNGKYUNKWAN UNIV	6
TEXAS A M UNIV	6
UNIV CALGARY	6
UNIV CAMERINO	6
UNIV CONNECTICUT HLTH	6
UNIV N CAROLINA	6
UNIV NEBRASKA MED CTR	6
UNIV PARIS 06	6
UNIV POLITECN MARCHE	6
UNIV SYDNEY	6
UNIV TOKYO	6
UNIV TWENTE	6
UNIV UTAH	6
UNIV WURZBURG	6

UNIV ZARAGOZA	6
UPMC UNIV PARIS 06	6
CATHOLIC UNIV DAEGU	5
CEDARS SINAI MED CTR	5
CREIGHTON UNIV	5
GRIFFITH UNIV	5
HACETTEPE UNIV	5
IOWA STATE UNIV	5
IRCCS GALEAZZI ORTHOPAED INST	5
JUSTUS LIEBIG UNIV GIESSEN	5
KENSEY NASH CORP	5
KOREA UNIV	5
KUMAMOTO UNIV	5
KURUME UNIV	5
KYUNGPOOK NATL UNIV	5
MCGILL UNIV	5
MRC ARTHRIT RES UK CTR INTEGRATED RES MUSCULOSKEL	5
NATL UNIV IRELAND GALWAY NUI GALWAY	5
NATL UNIV SINGAPORE HOSP	5
NATL YANG MING UNIV	5
NIBR	5
OSAKA HLTH SCI UNIV	5
OSIRIS THERAPEUT	5
PEKING UNIV THIRD HOSP	5
RIZZOLI ORTHOPAED INST	5
ROYAL COLL SURGEONS IRELAND	5
SAMSUNG MED CTR	5
TAIPEI VET GEN HOSP	5
TECH UNIV DRESDEN	5
TEL AVIV UNIV	5
TRINITY COLL DUBLIN	5
UNIV ALABAMA BIRMINGHAM	5
UNIV BIRMINGHAM	5
UNIV CANTABRIA	5
UNIV CATTOLICA SACRO CUORE	5
UNIV COLOGNE	5
UNIV GLASGOW	5
UNIV GOTHENBURG	5
UNIV HOSP	5
UNIV MED DENT NEW JERSEY	5
UNIV MISSOURI	5
UNIV NEW S WALES	5
UNIV OKLAHOMA	5
UNIV PORTO	5

UNIV S FLORIDA	5
UNIV SAO PAULO	5
UNIV TURIN	5
UNIV ULM	5
XIANGYA HOSP	5
ZUNYI MED UNIV	5
ACECR	4
ANKARA UNIV	4
ARMY MED UNIV	4
BEIHANG UNIV	4
BISPEBJERG HOSP	4
BROWN UNIV	4
CARNEGIE MELLON UNIV	4
CATHOLIC UNIV KOREA	4
CHILDRENS HOSP PITTSBURGH	4
CHINA MED UNIV HOSP	4
CHINA ORTHOPAED REGENERAT MED CORMED	4
CINCINNATI SPORTS MED ORTHOPAED CTR	4
CITY UNIV HONG KONG	4
DANKOOK UNIV	4
DREXEL UNIV	4
ECOLE POLYTECH FED LAUSANNE	4
FEINSTEIN INST MED RES	4
GUANGZHOU MED UNIV	4
GUANGZHOU UNIV CHINESE MED	4
HEIDELBERG UNIV	4
HULL YORK MED SCH	4
HUMBOLDT UNIV	4
INJE UNIV	4
IRAN UNIV MED SCI	4
IRCCS	4
JIKEI UNIV	4
LLR UNIV VET ANIM SCI	4
LOMA LINDA UNIV	4
LUDWIG BOLTZMANN INST EXPT CLIN TRAUMATOL	4
NAGASAKI UNIV	4
NARA MED UNIV	4
NATL RES CTR EQUINES	4
NATL RES INST CHILD HLTH DEV	4
OREGON STATE UNIV	4
POLISH ACAD SCI	4
POLITECN MILAN	4
PT GOVT ASSOCIATE LAB	4
QINGDAO UNIV	4
SHIRAZ UNIV	4

SIR CHARLES GAIRDNER HOSP	4
TAIPEI MED UNIV	4
UCONN HLTH	4
UNIV ADELAIDE	4
UNIV ALBERTA	4
UNIV BARI ALDO MORO	4
UNIV BASEL HOSP	4
UNIV CALIF IRVINE	4
UNIV COIMBRA	4
UNIV COLL HOSP	4
UNIV COLORADO	4
UNIV ESTADUAL PAULISTA	4
UNIV FED SAO PAULO	4
UNIV GEORGIA	4
UNIV GOTTINGEN	4
UNIV LYON 1	4
UNIV MINNESOTA	4
UNIV MONTREAL	4
UNIV NAPLES FEDERICO II	4
UNIV NOTTINGHAM	4
UNIV OTTAWA	4
UNIV PARMA	4
UNIV PORTSMOUTH	4
UNIV QUEENSLAND	4
UNIV ROMA LA SAPIENZA	4
UNIV ROMA TOR VERGATA	4
UNIV TEHRAN MED SCI	4
UNIV VIRGINIA HLTH SYST	4
UNIV ZURICH HOSP	4
URMIA UNIV	4
WARSAW UNIV LIFE SCI	4
WEILL CORNELL MED COLL	4
ZHEJIANG CHINESE MED UNIV	4
AJOU UNIV	3
ANACURA GRP	3
ASIA UNIV	3
ASST CTR SPECIALIST ORTOPED TRAUMATOL GAETANO PIN	3
BERLIN INST HLTH	3
BOSTON CHILDRENS HOSP	3
CARDIFF UNIV	3
CARDIOCTR TICINO	3
CATHOLIC UNIV	3
CENT INST RES BUFFALOES	3
CHANG GUNG MEM HOSP	3

CHINESE PEOPLES LIBERAT ARMY	3
CHONBUK NATL UNIV	3
CUNY CITY COLL	3
FAC VET MED	3
FIFA MED CTR EXCELLENCE	3
FLEXCELL INT CORP	3
GIFU UNIV	3
GOETHE UNIV FRANKFURT	3
GUANGDONG ACAD MED SCI	3
GUANGXI MED UNIV	3
HANYANG UNIV	3
HEBEI MED UNIV	3
HELMHOLTZ CTR INFECT RES HZI	3
HONG KONG POLYTECH UNIV	3
HOP ST ELOI	3
HOSP BRAGA	3
HOSP VITHAS SAN JOSE	3
HUNAN UNIV	3
INDIAN INST TECHNOL	3
INDIANA UNIV	3
INL INT IBERIAN NANOTECHNOL LAB	3
INNOVENT EV	3
IST ZOOPROFILATT SPERIMENTALE LOMBARDIA EMILIA	3
ISTANBUL UNIV	3
JINAN UNIV	3
JUNGWON UNIV	3
JUSTUS LIEBIG UNIV	3
KIDS RES INST	3
KOREA INST SCI TECHNOL	3
LEIBNIZ UNIV HANNOVER	3
LUND UNIV	3
MED UNIV WARSAW	3
MT SINAI HOSP	3
MT SINAI SCH MED	3
NANCY UNIV	3
NANTES UNIV HOSP	3
NATL CHENG KUNG UNIV	3
NATL INST DENT CRANIOFACIAL RES	3
NEW JERSEY REGENERAT INST	3
NEWCASTLE UNIV	3
NIPPON MED SCH	3
NORTH SICHUAN MED COLL	3
NUI GALWAY	3
PLA	3
POLITECN TORINO	3

QUEENS UNIV BELFAST	3
RENSSELAER POLYTECH INST	3
RICE UNIV	3
ROWAN UNIV	3
SAO PAULO STATE UNIV UNESP	3
SEOUL NATL UNIV HOSP	3
SHANGHAI 9TH PEOPLES HOSP	3
SORBONNE UNIV	3
SOUTHWEST UNIV	3
ST MARYS HOSP	3
STEMTECH GRP	3
STOWERS INST MED RES	3
TECH UNIV	3
TIKRIT UNIV	3
TOKAI UNIV	3
UNESP	3
UNIV CALIF BERKELEY	3
UNIV CHICAGO	3
UNIV DUISBURG ESSEN	3
UNIV DUSSELDORF	3
UNIV ELECT SCI TECHNOL CHINA	3
UNIV ESTADUAL CAMPINAS	3
UNIV FED MINAS GERAIS	3
UNIV GRANADA	3
UNIV HOSP MUNICH	3
UNIV HOSP MUNICH LMU	3
UNIV KENTUCKY	3
UNIV MED CTR REGENSBURG	3
UNIV MIAMI	3
UNIV NAT RESOURCES LIFE SCI BOKU	3
UNIV NEBRASKA	3
UNIV NORTH CAROLINA CHAPEL HILL	3
UNIV OREGON	3
UNIV PARIS EST	3
UNIV PAVIA	3
UNIV PERUGIA	3
UNIV PISA	3
UNIV POLITECN MADRID	3
UNIV ST JOSEPH	3
UNIV TENNESSEE	3
UNIV TEXAS AUSTIN	3
UNIV TEXAS HLTH SCI CTR HOUSTON	3
UNIV TOLEDO	3
UNIV TRENTO	3
UNIV TUBINGEN	3

UNIV ULSAN	3
UNIV VERONA	3
UNIV VITA SALUTE SAN RAFFAELE	3
UNIV WESTERN ONTARIO	3
UPMC	3
USC	3
VET AFFAIRS MED CTR	3
VIRGINIA POLYTECH INST STATE UNIV	3
WARSAW UNIV TECHNOL	3
WESTERN UNIV HLTH SCI	3
WUHAN GEN HOSP GUANGZHOU MIL REG	3
XISHAN PEOPLES HOSP	3
ZHEJIANG ACAD MED SCI	3
88TH HOSP PLA	2
AIR FORCE ENGN UNIV	2
AIRLANGGA UNIV	2
AKDENIZ UNIV	2
ALMA MATER STUDIORUM UNIV BOLOGNA	2
ANKARA ONCOL RES TRAINING HOSP	2
ARISTOTLE UNIV THESSALONIKI	2
ASSUT EUROPE SPA	2
AUSTRIAN ACAD SCI	2
AVANTEA SRL	2
BAOAN DIST PEOPLES HOSP SHENZHEN	2
BASKENT UNIV	2
BAYLOR COLL MED	2
BEIJING JISHUITAN HOSP	2
BEIJING MIL REG	2
BENGBU MED COLL	2
BERGMAN CLIN	2
BETH ISRAEL DEACONESS MED CTR	2
BIOCRUCES HLTH RES INST	2
BIOMED RES NETWORKING CTR BIOENGN BIOMAT NANOME	2
BIONAND	2
BIOSTAT RETRIEVER	2
BIOTIME INC	2
BST	2
CHA UNIV	2
CHANGZHOU 2 PEOPLES HOSP	2
CHILDRENS HOSP	2
CHILDRENS HOSP BOSTON	2
CHINA ORTHOPAED REGENERAT MED GRP CORMED	2
CHINESE ACAD AGR SCI	2
CHINESE ACAD MED SCI	2

CHONGQING FIRST PEOPLES HOSP	2
CHONGQING TRADIT CHINESE MED HOSP	2
CHONNAM NATL UNIV	2
CHU NANTES	2
CHUNG SHAN MED UNIV	2
CIBER BBN	2
CINCINNATI CHILDRENS HOSP	2
CLIN USP LA ESPERANZA	2
COMMUNITY TISSUE SERV	2
COMPLEJO HOSP UNIV A CORUNA	2
COORDINATED HLTH	2
CTR HOSP SAO JOAO	2
DALIAN MED UNIV	2
DALIAN MUNICIPAL CENT HOSP	2
DEAKIN UNIV	2
DEPT MECH AEROSP ENGN	2
DEPT ORTHOPAED	2
DEPT RIZZOLI RIT	2
DEPT SURG	2
DOKKYO MED UNIV	2
DOSHISHA UNIV	2
DUBLIN CITY UNIV	2
EMPA	2
ENTE OSPED CANTONALE	2
EOC	2
EQUINE VET PRACTICE DR SULS	2
ERASMUS MC	2
ESKISEHIR OSMANGAZI UNIV	2
EWHA WOMANS UNIV	2
EXPT ZOOPROPHYLACT INST UMBRIA MARCHE	2
FAC MED	2
FAC MED VET	2
FDN POLIAMBULANZA IST OSPED	2
FIRST HOSP JILIN UNIV	2
FLORIDA INST TECHNOL	2
FRED HUTCHINSON CANC RES CTR	2
FUJIAN MED UNIV	2
FUJIAN NORMAL UNIV	2
GEN HOSP CHINESE PEOPLES LIBERAT ARMY	2
GEORGETOWN UNIV	2
GUANGDONG MED UNIV	2
GWANGJU INST SCI TECHNOL	2
GWO XI STEM CELL APPL TECHNOL CO LTD	2
HALLYM UNIV	2
HANNAM UNIV	2

HANNOVER MED SCH MHH	2
HARVARD MIT DIV HLTH SCI TECHNOL	2
HARVARD STEM CELL INST	2
HEALTHBABY BIOTECH HONG KONG CO LTD	2
HELMHOLTZ CTR INFECT RES	2
HENAN PROV PEOPLES HOSP	2
HERMINIO OMETTO UNIV CTR UNIARARAS	2
HOP ST ANTOINE	2
HORMOZGAN UNIV MED SCI	2
HOSP BOLZANO	2
HOSP CLIN SAN CARLOS IDISSC	2
HOSP REFERENCIA LA EQUINA	2
HOSP UNIV RAMON CAJAL	2
HOSP WUHAN SPORTS UNIV	2
HUASHAN HOSP	2
INDIANA UNIV SCH MED NW	2
INGENERON INC	2
INHA UNIV	2
INSERM U1156	2
INST PASTEUR	2
INST SPORT EXERCISE HLTH	2
INT INST TRANSLAT MED	2
IRCCS IST ORTOPED RIZZOLI	2
ISF COLL PHARM	2
ISFAHAN UNIV MED SCI	2
ISFAHAN UNIV TECHNOL	2
IST ORTOPED GALEAZZI	2
IST ORTOPED RIZZOLI	2
IST ZOOPROFILATT SPERIMENTALE ABRUZZO MOLISE G	2
JAPAN AGCY MED RES DEV	2
JAPAN RACING ASSOC	2
JOHN COCHRAN VA MED CTR	2
KANAZAWA UNIV	2
KANSAI MED UNIV	2
KAOHSIUNG MED UNIV	2
KAOHSIUNG MED UNIV HOSP	2
KAROLINSKA UNIV HOSP HUDDINGE	2
KEIO UNIV	2
KEY LAB MUSCULOSKELETAL SYST DEGENERAT REGENERA	2
KING ABDULAZIZ UNIV	2
KINGS COLL LONDON	2
KOREA POLYTECH UNIV	2
KYOTO UNIV HOSP	2
LEIDEN UNIV	2

MAASTRICHT UNIV	2
MACQUARIE UNIV	2
MARIA SKLODOWSKA CURIE MEM CANC CTR	2
MARSHALL UNIV	2
MAX PLANCK INST MOL GENET	2
MED COLL GEORGIA	2
MED UNIV GRAZ	2
MED UNIV VIENNA	2
MERCK RES LABS	2
METU	2
MIN SHENG GEN HOSP	2
MRC	2
MT SINAI HLTH SYST	2
MUNICH UNIV APPL SCI	2
MYONGJI UNIV	2
N CAROLINA STATE UNIV	2
NANJING UNIV CHINESE MED	2
NANTES UNIV	2
NATL DEF MED COLL	2
NATL HLTH RES INST	2
NATL UNIV SINGAPORE SUZHOU	2
NEW JERSEY INST TECHNOL	2
NIAMSD	2
NIST	2
NOC NSF	2
NORFOLK NORWICH UNIV HOSP	2
NOVARTIS INST BIOMED RES	2
OKLAHOMA STATE UNIV	2
OLD DOMINION UNIV	2
OLVG WEST	2
ONZE LIEVE VROUW HOSP	2
ORTHOPADIE VIKTUALIENMARKT	2
OSAKA PREFECTURE UNIV	2
OSPED SAN GIOVANNI DIO RUGGI DARAGONA	2
OTTAWA HOSP	2
OTTO VON GUERICKE UNIV	2
PAIN MANAGEMENT CTR AMER	2
PARCO TECNOL PADANO	2
PEKING UNION MED COLL	2
PEOPLES LIBERAT ARMY GEN HOSP	2
PFERDEKLINK KIRCHHEIM	2
PINGHU SECOND PEOPLES HOSP	2
POHANG UNIV SCI TECHNOL POSTECH	2
RAMBAM MED CTR	2
REGENEXX LLC	2

ROYAL FREE LONDON NHS FDN TRUST HOSP	2
ROYAL LONDON HOSP	2
ROYAL ORTHOPAED HOSP	2
RUHR UNIV BOCHUM	2
S CHINA UNIV TECHNOL	2
SAARLAND UNIV	2
SALZBURG UNIV	2
SEMMELWEIS UNIV	2
SHAANXI PROV PEOPLES HOSP	2
SHANGHAI MED UNIV 2	2
SHAOXING PEOPLES HOSP	2
SHENYANG MIL AREA COMMAND	2
SHENZHEN PEOPLES HOSP	2
SHENZHEN UNIV	2
SHIRAZ UNIV MED SCI	2
SICHUAN COLL TRADIT CHINESE MED	2
SOUTH CHINA UNIV TECHNOL	2
SOUTHWEST HOSP	2
SPIRITO SANTO HOSP	2
ST LOUIS UNIV	2
ST VINCENT SHOULDER SPORTS CLIN	2
STANFORD HLTH CARE	2
STEMS MED CLIN	2
SUNY BUFFALO	2
SURG ENERGET INC	2
SWISS STEM CELL FDN	2
TAMPERE UNIV HOSP	2
TANGSHAN WORKERS HOSP	2
TECNOL MONTERREY	2
TIANJIN HOSP	2
TOKYO DENT COLL	2
TOKYO WOMENS MED UNIV	2
TOR VERGATA ROME UNIV	2
TRAUMA CTR MURNAU	2
TUFTS UNIV NEW ENGLAND MED CTR	2
UNIV APPL SCI TECHNIKUM WIEN	2
UNIV ARIZONA	2
UNIV ATHENS	2
UNIV AUCKLAND	2
UNIV AUSTRAL CHILE	2
UNIV AUTONOMA MADRID	2
UNIV BARI	2
UNIV BASEL	2
UNIV BASQUE COUNTRY	2
UNIV BELGRADE	2

UNIV BRITISH COLUMBIA	2
UNIV CALDAS	2
UNIV COLL LONDON HOSP	2
UNIV E ANGLIA	2
UNIV EAST ANGLIA	2
UNIV ERLANGEN NURNBERG	2
UNIV FED ABC UFABC	2
UNIV FED FLUMINENSE	2
UNIV FLORENCE	2
UNIV GRONINGEN	2
UNIV HEIDELBERG	2
UNIV HELSINKI	2
UNIV HOSP BALGRIST	2
UNIV HOSP SALZBURG	2
UNIV HOSP ZURICH	2
UNIV HOUSTON	2
UNIV IOANNINA	2
UNIV JIMEI	2
UNIV KEBANGSAAN MALAYSIA	2
UNIV KLINIKUM HEIDELBERG	2
UNIV LAUSANNE HOSP	2
UNIV LIMERICK	2
UNIV LONDON IMPERIAL COLL SCI TECHNOL MED	2
UNIV LORRAINE	2
UNIV MALAGA	2
UNIV MANITOBA	2
UNIV MED CTR	2
UNIV MELBOURNE	2
UNIV MILANO BICOCCA	2
UNIV MUNSTER	2
UNIV NANTES	2
UNIV NEBRASKA LINCOLN	2
UNIV NEW SOUTH WALES	2
UNIV NICE SOPHIA ANTIPOLIS	2
UNIV PACIFIC	2
UNIV PARIS 13	2
UNIV SAARLAND	2
UNIV SCI	2
UNIV SCI TECHNOL CHINA	2
UNIV SOUTHAMPTON	2
UNIV STRASBOURG	2
UNIV TABRIZ	2
UNIV TAMPERE	2
UNIV TECHNOL COMPIEGNE	2
UNIV TEHRAN	2

UNIV TEXAS	2
UNIV TEXAS HLTH SCI CTR SAN ANTONIO	2
UNIV TEXAS HOUSTON	2
UNIV TEXAS SOUTHWESTERN MED CTR DALLAS	2
UNIV VALENCIA	2
UNIV VERMONT	2
UNIV VET MED VIENNA	2
UNIV VIENNA	2
URMIA UNIV MED SCI	2
VA GREATER LOS ANGELES HEALTHCARE SYST	2
VIRGINIA COMMONWEALTH UNIV	2
WAKE FOREST SCH MED	2
WAKE FOREST UNIV	2
WENZHOU MED COLL	2
WESTERN MICHIGAN UNIV	2
WONKWANG UNIV	2
WRITTLE AGR COLL	2
WROCLAW UNIV TECHNOL	2
WYETH DISCOVERY RES	2
XIAMEN UNIV	2
YALE UNIV	2
YANGZHOU UNIV	2
ZHENGZHOU UNIV	2
1ST MIL CLIN HOSP	1
3D BIOPRINTING SOLUT	1
513 HOSP PLA	1
AALBORG UNIV	1
AARHUS UNIV	1
AARHUS UNIV HOSP	1
AB MED	1
AB MED SPA	1
ACAD ATHENS	1
ACAD CTR DENT AMSTERDAM	1
ACAD MED CTR	1
ACAD ORTHOPAED	1
ACAD ORTHOPAED GUANGDONG PROV	1
ACAD ORTHOPED	1
ACAD SCI CZECH REPUBLIC	1
ACAD SINICA	1
ACADEMIA	1
ADDENBROOKES HOSP	1
ADNAN MENDERES UNIV	1
ADV BIOTECHNOL INC	1
ADV SPINE PK AVE	1
ADVOCATE ILLINOIS MASONIC MED CTR	1

AFFILIATED HOSP CHENGDE MED COLL	1
AFFILIATED HOSP HUBEI PROV GOVT	1
AFFILIATED HOSP LUZHOU	1
AHVAZ JUNDISHAPUR UNIV MED SCI	1
AICHI MED UNIV	1
AIR FORCE GEN HOSP	1
AIZAWA HOSP	1
AJA UNIV MED SCI	1
ALAMO PINTADO EQUINE MED CTR	1
ALEXANDRA HOSP	1
ALL INDIA INST MED SCI	1
ALTA ORTHOPAED	1
AMBO UNIV	1
AMER BRITISH COWDRAY MED CTR	1
AMER ORTHOPAED SOC SPORTS MED	1
AMER UNIV BEIRUT	1
AMIL UNITEDHLTH GRP	1
AMRITA INST MED SCI	1
AMRITA VISHWA VIDYAPEETHAM	1
AMSTERDAM MED CTR	1
AN NAJAH NATL UNIV HOSP	1
ANAHUAC UNIV	1
ANDREWS INST	1
ANIKA THERAPEUT INC	1
ANKARA YILDIRIM BEYAZIT UNIV	1
AO CITTA SALUTE SCI TORINO	1
AO FDN	1
AO RES INST	1
AO RES INST DAVOS	1
AOU FEDERICO II NAPLES	1
ARAGON HLTH SCI INST	1
ARIZONA STATE UNIV	1
ARMY MIL MED UNIV	1
ARTHREX	1
ARTS METIERS PARISTECH	1
ASAHIKAWA MED UNIV	1
ASAN MED CTR	1
ASIA UNIV HOSP	1
ASKLEPIOS KLIN ST GEORG	1
ASKLEPIOS STADTKLIN	1
ASL VARESE	1
ASOCIAC INVEST IND TEXT AITEX	1
ASSOCIATE PT GOVT LAB	1
ASST GAETANO PINI	1
ATATURK TRAINING RES HOSP	1

ATHENA BIOMED INNOVAT	1
ATOS CLIN MUNICH	1
ATTIKON ANIM HOSP	1
ATTIKON UNIV GEN HOSP	1
ATTIKON UNIV HOSP	1
AUCKLAND CITY HOSP	1
AUDRAIN MED CTR	1
AUKAMM CLIN	1
AUSTRALIA PERTH BONE TISSUE BANK	1
AUSTRALIAN RES COUNCIL	1
AUSTRALIAN RES COUNCIL TRAINING CTR INNOVAT BIOEN	1
AUSTRIAN CLUSTER TISSUE ENGN	1
AUVA RES CTR	1
AUVA TRAUMA CTR VIENNA MEIDLING	1
AVICENNA RES INST	1
AZIENDA OSPED	1
AZIENDA OSPED MAGGIORE CARITA	1
AZIENDA SOCIO SANIT TERR CTR SPECIALIST ORTOPED T	1
AZIENDA SOCIO SANITARIA TERR	1
BAGCILAR TRAINING RES HOSP	1
BALTIMORE VA MED CTR	1
BARCELONA UNIV	1
BARTHOLOMEWS ROYAL LONDON HOSP	1
BARTS HLTH NHS TRUST	1
BARTS LONDON HOSP	1
BARTS LONDON NHS TRUST	1
BARTS LONDON QUEEN MARYS SCH MED DENT	1
BARUNSESANG HOSP	1
BAYLOR SCOTT WHITE TEXAS SPINE JOINT HOSP	1
BEAUMONT HLTH	1
BEAUMONT HLTH SYST	1
BEIGANG HOSP	1
BEIJING HOSP	1
BEIJING SPORT UNIV	1
BEIJING UNIV CHEM TECHNOL	1
BERLIN BRANDENBURG CTR REGENERAT THERAPIES	1
BERUFSGENOSSENSCHAFTLICHE UNIV KLIN BERGMANNSHEIL	1
BETA KLIN	1
BEZMIALEM VAKIF UNIV	1
BINZHOU PEOPLES HOSP	1
BIOACT SURG INC	1
BIOCANT BIOTECHNOL INNOVAT CTR	1
BIOENGN PROGRAM	1

BIOMAT NANOMED CIBER BBN	1
BIOMED CTR	1
BIOMEDICUM HELSINKI	1
BIOMET	1
BIOMIMET THERAPEUT INC	1
BIOSCAFFOLD INT PTE LTD	1
BIOTECHNOL INST IMASD	1
BIOTECHNOL INST MAS D 1	1
BIOVALDA HLTH TECHNOL INC	1
BK21 PLUS FUTURE BIOPHARMACEUT HUMAN RESOURCES TR	1
BOCA RATON REG HOSP	1
BONUS BIOGRP LTD	1
BOSTON SPORTS SHOULDER CTR	1
BRAGA GUIMARAES	1
BRAUNSCHWEIG UNIV TECHNOL	1
BRNO UNIV TECHNOL	1
BROAD INST HARVARD MIT	1
BROOKHAVEN NATL LAB	1
BROOMFIELD HOSP	1
BRUCE W CARTER VET AFFAIRS MED CTR	1
BTI BIOTECHNOL INST	1
BTI BIOTECHNOL INST IMASD	1
BUDAPEST UNIV TECHNOL ECON	1
BUKKYO UNIV	1
BULGARIAN ACAD SCI	1
BULINGTON EMERGENCY VET SPECIALISTS	1
BUNDANG JAESAENG HOSP	1
BURNHAM INST	1
BUTANTAN INST	1
C WAYNE MCILWRAITH TRANSLAT MED INST	1
CABA	1
CACTUS HEALTHCARE	1
CAIRO UNIV	1
CALIF INST BIOMED RES	1
CALIF STATE UNIV SACRAMENTO	1
CAMBRIDGE UNIV HOSP NHS FDN TRUST	1
CAMPUS BIOMED UNIV ROME	1
CANADIAN BLOOD SERV	1
CANC RES UK	1
CANGZHOU INTEGRATED TRADIT CHINESE WESTERN MED	1
CAPPAGH NATL ORTHOPAED HOSP	1
CARDENAL HERRERA CEU UNIV	1
CARDIOENERGETICS	1
CARDIOENERGETICS INC	1

CARE OF CAMIRE L	1
CARITAS KRANKENHAUS BAD MERGENTHEIM	1
CARLO POMA HOSP	1
CARMEL HOSP	1
CARNEGIE INST SCI	1
CAROLINA MED CTR	1
CAROLINAS MED CTR	1
CASE WESTERN RESERVE UNIV HOSP	1
CAVE CREEK EQUINE SURG IMAGING CTR	1
CELAVIE BIOSCI LLC	1
CELL ENGN ORIGIN	1
CELLCOTEC	1
CELLUMED CO LTD	1
CENT TAIWAN UNIV SCI TECHNOL	1
CEU UNIV	1
CHA BIO DIOSTECH CO LTD	1
CHANG GUNG UNIV MED COLL	1
CHANG GUNG UNIV SCI TECHNOL	1
CHANGCHUN UNIV CHINESE MED	1
CHANGPING DIST HOSP	1
CHANGZHI MED COLL	1
CHAPEL ALLERTON HOSP	1
CHARING CROSS WESTMINSTER MED SCH	1
CHARLES STURT UNIV	1
CHARLES UNIV PRAGUE	1
CHENGDU MIL GEN HOSP	1
CHENGDU UNIV TRADIT CHINESE MED	1
CHIBA UNIV	1
CHIBA UNIV HOSP	1
CHILDRENS HOSP BAMBINO GESU	1
CHILDRENS HOSP RES FDN	1
CHILDRENS MEM HOSP	1
CHILDRENS UNIV HOSP	1
CHINA ASTRONAUT RES TRAINING CTR	1
CHINA ORTHOPED REGENERAT MED CORMED	1
CHINA PEOPLES LIBERAT ARMY	1
CHINA REGENERAT MED CORMED	1
CHINA THREE GORGES UNIV	1
CHINESE MED ASSOC	1
CHINESE MED UNIV	1
CHIRURG TIERKLIN	1
CHON BUK NATL UNIV	1
CHONGQING ENGN RES CTR MICRONANO BIOMED MAT DEV	1
CHONGQING KEY LAB ORAL DIS BIOMED SCI	1

CHONGQING KEY LAB ULTRASOUND MOL IMAGING	1
CHONGQING NORMAL UNIV	1
CHONGQING UNIV MED SCI	1
CHRYSALIS BIOTECHNOL INC	1
CHU	1
CHU NANCY BRABOIS	1
CHUNG HWA UNIV	1
CHUNG SHAN MED UNIV HOSP	1
CHUNGNAM NATL UNIV	1
CHURCHILL HOSP	1
CHUV	1
CINCINNATI CHILDRENS HOSP MED CTR	1
CINCINNATI CHILDRENS HOSP RES FDN	1
CITY HOPE NATL MED CTR	1
CLIN CREU BLANCA	1
CLIN HORSES	1
CLIN ORTHOPAED EMERGENCY SURG	1
CLIN RADIOL OKLAHOMA	1
CLIN SPORT PARIS V	1
CLIN VET EQUINE PRACTICE	1
CMU	1
CNIC	1
CNRS UMR 7622	1
CNRS UMR 8104	1
COL MICHAEL J CRESCENZ VET ADM MED CTR	1
COLL MED	1
COLUMBUS CLIN	1
COMENIUS UNIV	1
COMPLEJO HOSP UNIV CANARIAS	1
COMPREHENS PAIN MANAGEMENT CTR	1
CONSELL CATALA ESPORT	1
CONSORZIO RIC SANIT CORIS	1
CONSORZIO RIC SANITARIA CORIS	1
CONSTRUCT ORTHOPED RES EDUC FDN	1
CORPORAL MICHAEL J CRESCENZ VA MED CTR	1
CORPORAL MICHAEL J CRESCENZ VET AFFAIRS MED CTR	1
CREAT BIOMOL INC	1
CREST JAPAN AGCY MED RES DEV	1
CRRL	1
CSIC	1
CSIRO	1
CTO HOSP	1
CTR ATUALIZACAO SAUDE	1
CTR CELLULAR MOL ENGN	1
CTR CHIRURG ORTHOPED SPORT	1

CTR EXCELLENCE BIOINFORMAT LIFE SCI	1
CTR EXPT RHEUMATOL	1
CTR HOSP POVOA VARZIM	1
CTR HOSP UNIV NANTES	1
CTR INVEST BIOMED RED BIOINGN BIOMAT NANOMED CI	1
CTR INVEST PRINCIPE FELIPE	1
CTR MED TEKNON	1
CTR MICROBIOROBOT SSSA	1
CTR MOL ORTHOPAED	1
CTR PAIN MANAGEMENT	1
CTR REGENERAT MED STEM CELL RES MFG LIVMEDCEL	1
CTR TRANSLAT BONE JOINT SOFT TISSUE RES	1
CTR TRAUMA	1
CTR UNIV CELSO LISBOA	1
CTR UNIV ICESP	1
CURTIN UNIV TECHNOL	1
CZECH TECH UNIV	1
DALHOUSIE UNIV	1
DALIAN UNIV	1
DALIAN UNIV TECHNOL	1
DANA FARBER CANC INST	1
DEF MED REHABIL CTR	1
DELEGAC CATALUNA	1
DEMOCRITUS UNIV THRACE	1
DEPT ANESTHESIOL	1
DEPT BIOENGN	1
DEPT BIOMED ENGN	1
DEPT CIVIL ENGN	1
DEPT INTERNAL MED RHEUMATOL	1
DEPT LAB MED PATHOL	1
DEPT MED MICROBIOL ID	1
DEPT MUSCULOSKELETAL DISORDERS	1
DEPT NANOENGN	1
DEPT NEURAL PAIN SCI	1
DEPT ORTHOPAED SURG	1
DEPT ORTHOPAED TRAUMATOL	1
DEPT ORTHOPED	1
DEPT ORTHOPED SURG	1
DEPT VET SCI ANIM HLTH PROD FOOD SAFETY	1
DEPUY ORTHOPAED INC	1
DIPRECA HOSP	1
DIV 01 SW RES INST	1
DIV PLAST RECONSTRUCT SURG	1
DOKUZ EYLUL UNIV	1

DONALD BARBARA ZUCKER SCH MED HOFSTRA NORTHWELL	1
DONCASTER ROYAL INFIRM	1
DONGGUAN INST FOOD DRUG CONTROL	1
DONGYING CITY PEOPLES HOSP	1
DR ALFEN ORTHOPAED SPINE CTR	1
DR ALFEN ORTHOPED SPINE CTR	1
DR ATTAMAN PLLC	1
DR BLAZEKS DIST HOSP	1
DR CHASBULLAH ABDULMADJID GEN HOSP	1
DR MOSHIRI VET CLIN	1
DR SOERADJI TIRTONEGORO GEN HOSP	1
DR SOETOMO GEN HOSP	1
DRK BLUTSPENDEDIENST BADEN WURTTEMBERG HESSEN	1
DUKE NUS MED SCH	1
DZHK GERMAN CTR CARDIOVASC RES	1
EAST COAST VET IMAGING INC	1
EASTERN VIRGINIA MED SCH	1
ECOLE NATL VET	1
ECOLE NATL VET ALFORT	1
ECOLE POLYTECH	1
EDITH COWAN UNIV	1
EDUARDO ANITUA FDN BIOMED RES	1
EDWARD HINES JR VA HOSP	1
EHIME UNIV	1
EIT WROCLAW RES CTR	1
ELISABETH BRUYERE HOSP	1
EMBODY	1
EMORY ORTHOPAED SPINE CTR	1
EMORY ORTHOPAED SPORTS SPINE	1
ENCELL CO LTD	1
ENGN TECHNOL CTR TISSUE ENGN XIAN	1
EQUI VET CLIN HORSES	1
EQUINE BREEDING STUD	1
EQUINE CLIN DE WATERMOLEN	1
EQUINE CLIN MUHLEN	1
EQUINE DIAGNOST CTR	1
EQUINE HOSP	1
EQUINE VET PRATICE DR SULS	1
EQUITOM EQUINE HOSP	1
ERCIYES UNIV	1
ESA	1
ESCUELA TECN SUPER INGN IND UNED	1
ETHRIS GMBH	1
EUROPEAN COMMISS	1

EUROPEAN INST EXCELLENCE TISSUE ENGN REGENERAT	1
EUROPEAN UNIV CYPRUS	1
EXPT ZOOPROFILACT INST SICILY A MIRRI	1
FAC HLTH MED NURSING BEHAV SCI	1
FAC HOSP BRNO	1
FAC MED UNIV	1
FAC VET	1
FACTORSTEM LTD	1
FDN AVANTEA	1
FDN CARDIOCTR TICINO	1
FDN EDUARDO ANITUA	1
FDN GARCIA CUGAT	1
FDN JIMENEZ DIAZ	1
FDN POLIAMBULANZA	1
FDN POLIAMBULANZA IST OSPEDALIERO	1
FDN RES TECHNOL HELLAS	1
FDN RIMED	1
FDN STEM CELL RES	1
FED UNIV PAMPA	1
FED UNIV SAO CARLOS UFSCAR	1
FED UNIV SAO PAULO UNIFESP	1
FETHARD EQUINE HOSP	1
FIDIA ADV BIOPOLYMERS	1
FIDIA FARMACEUT SPA	1
FIOCRUZ MINAS	1
FIRST MIL MED UNIV	1
FIRST PEOPLES HOSP GUANGYUAN	1
FIRST PEOPLES HOSP HUZHOU	1
FIRST PEOPLES HOSP LIANYUNGANG	1
FIRST PEOPLES HOSP YONGKANG	1
FLINDERS UNIV S AUSTRALIA	1
FLORIDA ORTHOPED FOOT ANKLE CTR	1
FMU	1
FOOTBALL CLUB BARCELONA	1
FOOYIN UNIV	1
FORENS RES ANAL	1
FORSCHUNGSINST LEDER KUNSTSTOFFBAHNEN FILK	1
FRAUNHOFER INST TOXICOL AEROSOL RES	1
FREI UNIV BERLIN	1
FRIEDRICH ALEXANDER UNIV ERLANGEN NURNBERG	1
FUJIAN PROV HOSP	1
FUKUSHIMA UNIV	1
FUN JEN CATHOLIC UNIV	1
G DANNUNZIO FDN ITAB	1
GALEAZZI ORTHOPAED INST	1

GALLANT PET INC	1
GEMMA PROTOTIPI	1
GEN HOSP CHINESE PEOPLES ARMED POLICE FORCE	1
GEN HOSP CHINESE PLA	1
GEN HOSP PLA	1
GENE CELL INC	1
GENESIS PAIN SPECIALIST	1
GENEVA UNIV HOSP	1
GENZYME TISSUE REPAIR INC	1
GEORGE WASHINGTON UNIV	1
GEORGIA INST TECH	1
GEORGIA REGENTS UNIV	1
GEORGIA TECH	1
GEORGIA TECH EMORY UNIV	1
GERMAN ARMED FORCES HOSP ULM	1
GERMAN RED CROSS BLOOD DONAT SERV	1
GERMAN RES CTR BIOTECHNOL GBF	1
GERMAN SPORT UNIV COLOGNE	1
GESELL BIOTECHNOL FORSCH MBH	1
GLASGOW CALEDONIAN UNIV	1
GLASGOW ORTHOPAED RES INITIAT GLORI	1
GLOBAL STEM CELL TECHNOL	1
GLOBAL STEM CELL TECHNOL ANACURA GRP	1
GOLD COAST UNIV HOSP	1
GOLDEN JUBILEE NATL HOSP	1
GOTHENBURG UNIV	1
GOVIND BALLABH PANT UNIV AGR TECHNOL	1
GR CONSULTING INC	1
GRAZ UNIV TECHNOL	1
GREAT ORMOND ST HOSP SICK CHILDREN	1
GREEN CROSS WELLBEING CORP	1
GREENVILLE HLTH SYST	1
GUANGZHOU COMMAND	1
GUANGZHOU MIL AREA WUHAN	1
GUANGZHOU MIL REG	1
GUANGZHOU UNIV TRADIT CHINESE MED	1
GUIZHOU PROV PEOPLES HOSP	1
GUNMA UNIV	1
GUYS ST THOMAS HOSP	1
GUYS ST THOMASS UNIV HOSP	1
GUYUAN PEOPLES HOSP	1
GWO XI STEM CELL APPL TECHNOL	1
HADASSAH HEBREW UNIV MED CTR	1
HADASSAH MED CTR	1
HAFFKINE INST	1

HAINAN PROV NONGKEN SANYA HOSP	1
HAINAN UNIV	1
HANEUL ORTHOPED HOSP	1
HANGZHOU TCM HOSP	1
HANOVER MED SCH MHH	1
HARBIN CHILDREN HOSP	1
HARRISON INT PEACE HOSP	1
HARVARD SCH DENT MED	1
HCPA	1
HEALTHCARE TECHNOL INST	1
HEBEI UNIV ENGN	1
HEILONGJIANG PROV ACAD MED SCI	1
HEILONGJIANG PROV HOSP	1
HELMHOLTZ ZENTRUM BERLIN MAT ENERGIE	1
HELSINKI UNIV HOSP	1
HERZ JESU KRANKENHAUS	1
HESSING STIFTUNG	1
HESSING STIFTUNG AUGSBURG	1
HICKORY VET HOSP	1
HINSDALE ORTHOPAED	1
HIRANANDANI HOSP	1
HIROSHIMA PREFECTURAL REHABIL CTR	1
HLTH RES INST ARAGON IIS ARAGON	1
HMT HIGH MED TECHNOL AG	1
HOAG HOSP	1
HOFSTRA NORTHWELL HLTH	1
HOKKAIDO UNIV HOSP	1
HOKUSHIN ORTHOPAED HOSP	1
HONG KONG UNIV SCI TECHNOL	1
HOP BICETRE	1
HOP HENRI MONDOR	1
HOP PAUL BROUSSE	1
HOP PRIVE LOUVIERE	1
HOP ST ELOI INM	1
HOP TROUSSEAU	1
HOP UNIV CROIX ROUSSE	1
HOP XAVIER AMOZAN	1
HOSP BOAVISTA	1
HOSP CLIN MONTEVIDEO	1
HOSP CLIN SAN CARLOS	1
HOSP CLIN UNIV VIRGEN ARRIXACA	1
HOSP COSTA DEL SOL	1
HOSP CRUCES	1
HOSP ERNST VON BERGMANN	1
HOSP GEN UNIV GREGORIO MARANON	1

HOSP GROSSHADERN	1
HOSP HENRI MONDOR	1
HOSP JUAN RAMON JIMENEZ	1
HOSP LA PAZ	1
HOSP LUZHOU	1
HOSP MAE DEUS	1
HOSP MIL PEDRO V	1
HOSP PRIVADO BRAGA	1
HOSP QUIRONSALUD	1
HOSP SALERNO	1
HOSP SAO JOAO	1
HOSP SARAH SALVADOR	1
HOSP SICK CHILDREN	1
HOSP SPECIAL SURG FLORIDA	1
HOSP SPECIAL SURG HSS	1
HOSP TORREJON	1
HOSP UNIV CLIN SAN CARLOS MADRID	1
HOSP UNIV DONOSTIA	1
HOSP UNIV KEBANGSAAN MALAYSIA	1
HOSP UNIV LA PAZ	1
HOSP UNIV LA PRINCESA	1
HOSP UNIV MARQUES DE VALDECILLA	1
HOSP UNIV PENN	1
HOSP UNIV POLITECN LA FE	1
HOSP UNIV RAMON Y CAJAL	1
HOSP UNIV VIRGEN NIEVES	1
HOSP VALLE DE HEBRON	1
HOSPTIAL AUTHOR	1
HOWARD UNIV	1
HUBEI HANCHUAN PEOPLES HOSP	1
HUBEI PROV KEY LAB DEV ORIGINATED DIS	1
HUMANITAS RES HOSP	1
HUMANITAS SAN PIO X	1
HUNAN NORMAL UNIV	1
HUNGARIAN ACAD SCI	1
HYOGO COLL MED	1
IBB	1
IBPS DEV BIOL LAB	1
ICAN	1
ICAR INDIAN VET RES INST	1
ICOSS	1
ICVS	1
ICVS 3BS	1
ICVS 3BS PT ASSOCIATE LAB	1
ICVS 3BSPT GOVT ASSOCIATE LAB	1

IDEXX LABS	1
IFR	1
IGEA SPA	1
IGEA SPA CLIN BIOPHYS	1
IIT	1
IM SECHENOV FIRST MOSCOW STATE MED UNIV	1
IMAGINE INST	1
IMPERIAL COLL LONDON	1
INCLIVA BIOMED RES INST	1
IND TECHNOL RES INST	1
INDIAN INST TECHNOL GUWAHATI	1
INDIAN VET RES INST	1
INDIAN VET RES INST IZATNAGAR	1
INDIANA UNIV SCH MED	1
INEFC	1
INGENERON	1
INGENERON GMBH	1
INNOVHUB SSI	1
INOVARION	1
INSERM 1094	1
INST APPAREIL LOCOMOTEUR NOLLET	1
INST BIOL CHEM	1
INST BIOMED HLTH SCI	1
INST BIOTECHNOL	1
INST BONE CARTILAGE	1
INST CALOT	1
INST CHIRURG ORTHOPED SPORT ICOS 13	1
INST COCHIN	1
INST CUGAT	1
INST ENGN MED	1
INST ENT	1
INST G CAPORALE	1
INST INVEST BIOSANITARIA IBS	1
INST INVEST SANITARIA FDN JIMENEZ DIAZ	1
INST INVEST SANITARIA HOSP CLIN SAN CARLOS IDISSC	1
INST LIFE SCI	1
INST MOL CELL BIOL	1
INST MURCIANO INVEST DESARROLLO AGR ALIMENTAR	1
INST MUSCULOSKELETAL ANAL RES THERAPY	1
INST NACL CIENCIAS MED NUTR SALVADOR ZUBIRAN	1
INST NACL REHABIL LUIS GUILLERMO IBARRA IBARRA	1
INST ONCOL	1
INST ORTHOPED	1
INST PESQUISAS CELULAS TRONCO	1
INST RECH BIOMAT BIOTECHNOL	1

INST REGENERAT MED	1
INST SCI TECHNOL CERAM	1
INST TECHNOL	1
INST TECNOL ARAGON ITAINNOVA	1
INST TEXT TECHNOL PROC ENGN	1
INST TISSUE ENGN REGENERAT MED	1
INSTM	1
INSTM NATL INTERUNIV CONSORTIUM MAT SCI TECHNOL	1
INT CONSULTANCY BLOOD COMPONENTS QUAL SAFETY IMPR	1
INT MED UNIV	1
INT NEUROSCI INST	1
INT OLYMP COMM MED COMMISS	1
INT SOC CELL THERAPY	1
INVERNO MONTELEONE	1
IRANIAN ACAD CTR EDUC CULTURE RES ACECR	1
IRANIAN FOOD DRUG ADM	1
IRCCS INST ORTOPED RIZZOLI	1
IRCCS IST CLIN HUMANITAS	1
IRCCS ORTHOPED INST GALEAZZI	1
IRCCS RIZZOLI ORTHOPED INST	1
IRCCS SAN RAFFAELE PISANA	1
IRCCS SAN RAFFAELE SCI INST	1
IRRC GALEAZZI ORTHOPAED INST	1
ISAR KLINIKUM	1
ISME	1
ISOTIS NV	1
IST ITALIAN TECNOL	1
IST ITALIANO TECNOL	1
IST NAZL STAT ISTAT	1
IST ORTOPED GALEAZZI IRCCS	1
IST OSPED	1
IST SUPER SANITA	1
IST ZOOPROFILATT SPERIMENTALE ABRUZZO MOLISE GI	1
IST ZOOPROFILATT SPERIMENTALE LAZIO TOSCANA M A	1
IST ZOOPROFILATT SPERIMENTALE VENEZIE	1
IST ZOOPROFILATTICO SPERIMENTALE ABRUZZO MOLISE	1
ISTANBUL TECH UNIV	1
IWAMIZAWA KOJIN KAI HOSP	1
IZMIR DEMOCRACY UNIV	1
IZSLER	1
JACKSON LAB	1
JAGIELLONIAN UNIV	1
JAPAN COMMUNITY HLTH CARE ORG	1
JAPAN LABOUR HLTH WELF ORG	1

JAPAN SCI TECHNOL AGCY	1
JAPAN SCI TECHNOL CORP	1
JAPAN SELF DEF FORCE HOSP YOKOSUKA	1
JAPAN SOC PROMOT SCI	1
JASLOK HOSP RES CTR	1
JERRY L PETTIS MEM VA MED CTR	1
JI NAN UNIV	1
JIANGSU UNIV	1
JICHI MED UNIV	1
JILIN BIOMED POLYMERS ENGN LAB	1
JINAN CENT HOSP	1
JINMEN 2 PEOPLES HOSP	1
JL PETTIS MEM VA MED CTR	1
JOHN RADCLIFFE HOSP	1
JOHNS HOPKINS SCH MED	1
JONSSON ENGN CTR	1
JORDAN UNIV SCI TECHNOL	1
JULIUS MAXIMILIANS UNIV	1
KAGOSHIMA UNIV	1
KAISER PERMANENTE	1
KAISER PERMANENTE SAN DIEGO	1
KANTONSSPITAL WINTERTHUR	1
KAPLAN HOSP	1
KAPODISTRIAN UNIV ATHENS	1
KAROL MARCINKOWSKI MED UNIV	1
KASETSART UNIV	1
KATHOLIEKE UNIV LEUVEN	1
KAVLI INST BIONANO SCI TECHNOL	1
KAYAL ORTHOPAED CTR	1
KAZAN FED UNIV	1
KECK SCH MED USC	1
KEMILEW STEM CELLS ANIM CO	1
KERRY REHABIL MED RES INST	1
KEY LAB TISSUE ENGN REGENERAT MED ZHEJIANG PROV	1
KHALSA COLL VET SCI	1
KHARAZMEI UNIV	1
KING ABDULAZIZ UNIV HOSP	1
KING SAUD UNIV	1
KINGSTON HOSP NHS TRUST	1
KINKI UNIV	1
KIRCHHEIM ALTANO GMBH	1
KLINIKUM BOGENHAUSEN	1
KLINIKUM INGOLSTADT	1
KLINIKUM LANDSBERG LECH	1
KOBE KAISEI HOSP	1

KOBE ROSAI HOSP	1
KOC UNIV HOSP	1
KOCAELI UNIV	1
KOCHI UNIV	1
KOGAKUIN UNIV	1
KOLLING INST MED RES	1
KOREA BASIC SCI INST	1
KOREA INST SCI TECHNOL KIST	1
KOREA RES INST CHEM TECHNOL	1
KOREA UNIV SCI TECHNOL UST	1
KU MED CTR	1
KUMAMOTO UNIV HOSP	1
KYOWA HAKKO KIRIN	1
KYOWA HAKKO KIRIN KOREA	1
KYUNGPOOK NATL UNIV HOSP	1
KYUSHU UNIV	1
LA TOUR HOSP	1
LABEO FRANK DUNCOMBE	1
LAM BIOTECHNOL SA	1
LANKENAU INST MED RES	1
LANZHOU MIL COMMAND REG	1
LE REVE REGENERAT WELLNESS	1
LEEDS DENT INST	1
LEHIGH VALLEY HLTH NETWORK	1
LEIBNIZ INST POLYMERFORSCH DRESDEN EV IPF	1
LENOX HILL HOSP	1
LGI	1
LI KA SHING CTR	1
LIFEMAP SCI	1
LIOAD	1
LITHUANIAN UNIV HLTH SCI	1
LIV HOSP	1
LOMA LINDA MED UNIV	1
LONG ISL JEWISH MED CTR	1
LONGGANG ENT HOSP	1
LOUIS STOKES CLEVELAND VET AFFAIRS MED CTR	1
LOWER SAXONY CTR BIOMED ENGN IMPLANT RES DEV NI	1
LOZANO BLESA UNIV CLIN HOSP	1
LSU HLTH SCI CTR	1
LSU SCH MED	1
LUDWIG MAXIMILIAN UNIV MUNICH	1
LUDWIG MAXIMILIANS UNIV LMU	1
LUOHE MED COLL	1
LUZERNER KANTONSSPITAL	1
MACAU UNIV SCI TECHNOL	1

MACKAY MEM HOSP	1
MAHIDOL UNIV	1
MAIMONIDES HOSP	1
MAIMONIDES INST BIOMED RES CORDOBA IMIBIC	1
MAINLAND ACUPUNCTURE CTR	1
MAINSTREAM CONSULTING	1
MANLY HOSP	1
MARIA SKLODOWSKA CURIE INST	1
MARQUETTE UNIV	1
MAT SCI ENGN PROGRAM	1
MATER ADULT HOSP	1
MATER HLTH SERV	1
MATER MED RES INST	1
MATER RES INST UNIV QUEENSLAND	1
MATERN CHILD CARE HOSP PINGSHAN DIST	1
MAX BERGMANN CTR BIOMAT DRESDEN	1
MAX BIEDERMANN INST BIOMECH	1
MAX PLANCK INST COLLOIDS INTERFACES	1
MAX PLANCK INST HEART LUNG RES	1
MAX PLANCK INST MOL GENET MPI	1
MAYO CLIN ROCHESTER	1
MCDAVITT VET CLIN	1
MCGOWAN INST REGENERAT MED	1
MED SCH HANNOVER MHH	1
MED SPECTRUM TWENTE HOSP	1
MED SPORT 2000 SRL	1
MED UNIV INNSBRUCK	1
MED UNIV LUBECK	1
MED UNIV S CAROLINA	1
MED UNIV SOUTH CAROLINA	1
MED UNIV VARNA	1
MED UNIV WROCLAW	1
MEDIPOLE	1
MEDSTAR GEORGETOWN UNIV HOSP	1
MEDSTAR WASHINGTON HOSP	1
MEDWAY NHS FDN TRUST	1
MEIJI UNIV	1
MENLO SPORTS MED	1
MERLN INST TECHNOL INSPIRED REGENERAT MED	1
MESA INST NANOTECHNOL	1
MESENCHYMAL STEM CELL GRP	1
METU COE BIOMAT TISSUE ENGN	1
METU CTR EXCELLENCE BIOMAT TISSUE ENGN	1
MHH	1
MICHIGAN STATE UNIV	1

MID CTY ORTHOPAED SURG SPORTS MED	1
MIDDLE E TECH UNIV	1
MIDDLE EAST TECH UNIV	1
MIDDLEMORE HOSP	1
MIDWEST ORTHOPAED RUSH	1
MIL INST MED	1
MIMEDX GRP INC	1
MING CHI UNIV TECHNOL	1
MING MED SERV SDN BHD	1
MINIST EDUC	1
MINIST HLTH RUSSIAN FEDERAT	1
MIRIAM HOSP	1
MISSISSIPPI STATE UNIV	1
MISSOURI UNIV SCI TECHNOL	1
MIYAZAKI UNIV	1
MONFARED NIAKI ARMY HOSP	1
MONTPELLIER UNIV HOSP	1
MOSCOW SECHENOV MED UNIV	1
MOSCOW STATE ACAD VET MED BIOTECHNOL	1
MOUSE ANIM PATHOL LAB MAPLAB FONDAZIONE FILARET	1
MT SINAI MED CTR	1
MULTIDISCIPLINARY HOSP	1
MYOMICS INC	1
MYONGJI HOSP	1
MYRIAD GENET INC	1
N SHORE UNIV HOSP LIJ	1
NAGOYA UNIV GRAD SCH MED	1
NALGAE HOSP	1
NANFANG MED UNIV	1
NANJING JINLING HOSP	1
NANJING NORMAL UNIV	1
NANKAI UNIV	1
NANOTECHNOL REGENERAT MED COMMERCIALIZAT CTR LT	1
NANTONG MATERNAL CHILD HLTH HOSP	1
NATIONWIDE CHILDRENS HOSP	1
NATL BLOOD SERV	1
NATL CHUNG HSING UNIV	1
NATL CTR NEUROL PSYCHIAT	1
NATL CTR TISSUE CELL BANKING	1
NATL DEF MED CTR	1
NATL DEFENSE MED COLL	1
NATL ENGN RES CTR NANOTECHNOL	1
NATL ENGN RES CTR TISSUE RESTORAT RECONSTRUCT	1
NATL HOSP NORWAY	1

NATL HOSP ORG IOU NATL HOSP	1
NATL ILAN UNIV	1
NATL INST BIOL STAND CONTROLS	1
NATL INST CHILD HLTH DEV	1
NATL INST DENTAL CRANIOFACIAL RES	1
NATL INST GENET	1
NATL KAPODISTRIAN UNIV ATHENS	1
NATL NAGASAKI CENT HOSP	1
NATL POLICE HOSP	1
NATL RES CTR REHABIL TECH AIDS	1
NATL SPORT ACAD VASIL LEVSKI	1
NATL TAIWAN UNIV COLL MED	1
NATL UNIV HLTH SYST	1
NATL UNIV IRELAND UNIV COLL GALWAY	1
NAVAL GEN HOSP PLA	1
NAVAL MED RES CTR	1
NDORMS	1
NEOTHERIX LTD	1
NEUROSCI RES CTR LLC	1
NEW BOLTON CTR	1
NEW ENGLAND BAPTIST HOSP	1
NEW JERSEY REGENERAT MED INST	1
NEW YORK GIANTS	1
NEW YORK INST TECHNOL	1
NEW YORK PRESBYTERIAN HOSP	1
NEW YORK R D CTR TRANSLAT MED THERAPEUT INC	1
NEWMARKET EQUINE HOSP	1
NIFE LOWER SAXONY CTR BIOMED ENGN IMPLANT RES D	1
NIHR BIOMED RES CTR	1
NIHR UNIV COLL LONDON HOSP	1
NIIGATA MEIKUN HIGH SCH	1
NIMH	1
NINGBO 2 HOSP	1
NIPPON MED COLL HOSP	1
NIT	1
NORMANDIE UNIV	1
NORTH CHINA UNIV SCI TECHNOL	1
NORTH SHORE HOSP	1
NORTH SHORE LIJ HLTH SYST	1
NORTH SICHUAN COLL MED	1
NORTHEASTERN UNIV	1
NORTHERN SYDNEY LOCAL HLTH DIST	1
NORTHSHORE UNIV HEALTHSYST	1
NORTHWELL HLTH	1
NORTHWEST UNIV	1

NORTHWEST UNIV XIAN	1
NORTHWESTERN POLYTECH UNIV	1
NORWEGIAN SCH SPORT SCI	1
NOVAGENIT SRL	1
NOVARTIS RES FDN	1
NPIMR	1
NPIMR Y3	1
NUFFIELD ORTHOPAED CTR	1
NUMARES AG	1
NW ORTHOPAED INST	1
OCEAN UNIV CHINA	1
OCEANOG GRP PARQUES REUNIDOS	1
ODENSE UNIV HOSP	1
OFZ ORTHOPAD FACHZENTRUM WEILHEIM	1
OITA UNIV	1
OKINOSU HOSP	1
OLVG	1
OREGON EQUINE	1
ORGAN TECHNOL INC	1
ORGANOGENESIS	1
ORIENTAL BIO GRP	1
ORTHOPAED CTR OCON	1
ORTHOPAED HOSP GUANGDONG PROV	1
ORTHOPAED HOSP RES CTR	1
ORTHOPAED OBERLAND	1
ORTHOPAED RES LABS	1
ORTHOPAED SPECIALISTS OS	1
ORTHOPAED SURG CTR OCC	1
ORTHOPAED UNIV HOSP HEIDELBERG	1
ORTHOPED UNIV HOSP	1
ORTHOSOUTH SURG CTR	1
ORTHOSPORTS	1
ORVIT CLIN	1
OSAKA CITY UNIV	1
OSAKIDETZA	1
OSSACUR AG	1
OTR3 CO	1
OTTAWA HOSP RES INST	1
OU WB SCH MED	1
OUR LADY LOURDES HOSP	1
OXFORD BIOMAT LTD	1
PAIN RELIEF DAYTON	1
PALO ALTO HEALTHCARE SYST	1
PALO ALTO MED FDN	1
PALO ALTO VET AFFAIRS HLTH CARE CTR	1

PARACELSUS MED UNIV SALZBURG	1
PARACELSUS PRIVATE MED UNIV	1
PARIS 7 UNIV	1
PARIS LODRON UNIV SALZBURG	1
PASTEUR INST IRAN	1
PAUL EHRLICH INST	1
PAULISTA UNIV UNIP	1
PEAK ORTHOPED SPINE	1
PEKING UNIV SCH HOSP STOMATOL	1
PELE PEQUENO PRINCIPE INST	1
PENINSULA ORTHOPAED RES INST	1
PENN HOSP	1
PENN STATE COLL MED	1
PENN STATE UNIV COLL MED	1
PEOPLES HOSP DONGPING CTY	1
PEOPLES HOSP LEIZHOU	1
PEOPLES LIBERAT ARMY	1
PGIMER	1
PHARMAEXCEED SRL	1
PHILADELPHIA VA MED CTR	1
PHILADELPHIA VET AFFAIRS MED CTR	1
PITTSBURGH TECHNOL CTR	1
PLAST SURG HOSP	1
PMOI	1
POHANG UNIV SCI TECHNOL	1
POLICE ACAD	1
POLICLIN GB ROSSI	1
POLICLIN SAN DONATO	1
POLICLIN SCOTTE	1
POLYTECH MONTREAL	1
POLYTECH UNIV MARCHE	1
POLYTECHN UNIV MARCHE	1
PONTIFICAL CATHOLIC UNIV PARANA PUCPR	1
POSIT UNIV UP	1
POZNAN UNIV MED SCI	1
PRESTIGE BIORES PTE LTD	1
PRESTO	1
PRIME PODIATRY	1
PRINCESS ROYAL UNIV HOSP	1
PROXY BIOMED	1
PUAI HOSP	1
PURE SPORTS MED CLIN	1
QSPORTSMEDICINE	1
QUEEN ELIZABETH HOSP BIRMINGHAM	1
QUEEN VICTORIA HOSP	1

QUEENS UNIV	1
QUEENS VET SCH	1
QUT	1
RABIN MED CTR	1
RACECOURSE VET SURG CLIN	1
RADIOL RES CONSULTAT	1
RAYMOND BEVERLY SACKLER CTR BIOMED BIOL PHYS	1
RCSI	1
REABILITA CLIN	1
RED CROSS BLOOD TRANSFUS SERV NORTH EAST	1
REG UNIV HOSP MALAGA	1
REGENERAT ORTHOPED SPORTS MED	1
REGENERAT ORTHOPED SPORTS MED INST	1
REGENERAT SCI LLC	1
REGENEUS PTY LTD	1
REGINA ELENA INST CANC RES	1
REHABIL CLIN YAMAGUCHI	1
RES CTR	1
RES CTR AUVA	1
RES CTR TRAUMATOL	1
RES RADIOL RES CONSULTAT	1
RHEIN WESTFAL TH AACHEN	1
RHODE ISL HOSP	1
RI MED FDN	1
RICHARD L ROUDEBUSH VA MED CTR	1
RIKEN	1
ROHAN GENE CELL TECH	1
ROMED KLINIKUM	1
ROPBERT WOOD JOHNSON MED CTR	1
ROTHMAN INST	1
ROTHMAN INST ORTHOPAED	1
ROYAL CHILDRENS HOSP	1
ROYAL FREE HOSP	1
ROYAL FREE HOSP HAMPSTEAD NHS TRUST	1
ROYAL FREE NHS TRUST HOSP	1
ROYAL LIVERPOOL UNIV HOSP	1
ROYAL MARSDEN NHS FDN TRUST	1
ROYAL MELBOURNE HOSP	1
ROYAL NATL ORTHOPAED HOSP NHS TRUST	1
ROYAL NATL ORTHOPAED HOSP TRUST	1
ROYAL NATL THROAT NOSE EAR HOSP	1
ROYAL UNIV HOSP	1
RUISI BIOMAT LTD CO	1
RURAL DEV ADM	1
RUSH PRESBYTERIAN ST LUKES MED CTR	1

RUSSIAN ACAD SCI	1
RUTGERS SCH BIOMED HLTH SCI	1
RUTGERS SCH MED	1
RWTH AACHEN UNIV CLIN	1
S AFRICAN MED COUNCIL	1
SAGA UNIV	1
SAHLGRENS UNIV HOSP	1
SAHLGRENSKA UNIV HOSP MOLNDAL	1
SALK INST BIOL STUDIES	1
SAMUEL MERRITT UNIV	1
SAMUMED LLC	1
SAN FRANCISCO VA MED CTR	1
SAN FRANCISCO VET AFFAIRS HEALTHCARE SYST	1
SAN FRANCISCO VET AFFAIRS HLTH CARE SYST	1
SAN MARTINO HOSP	1
SAN PABLO CEU UNIV	1
SANFORD CONSORTIUM REGENERAT MED	1
SANFORD HLTH	1
SANFORD ORTHOPED SPORTS MED FARGO	1
SANFORD ORTHOPED SPORTS MED SIOUX FALLS	1
SANFORD RADIOL CLIN	1
SANTA MONICA ORTHOPAED SPORTS MED GRP	1
SANTO SPIRITO HOSP	1
SARAH HOSP	1
SARKEYS ENERGY CTR	1
SCH BIOL SCI	1
SCH BIOMED ENGN SCI	1
SCH MED SURG DENT	1
SCHULTHESS CLIN	1
SCRIPPS CLIN	1
SCRIPPS CTR ORGAN CELL TRANSPLANTAT	1
SCUOLA SUPER SANT ANNA	1
SE UNIV	1
SEATTLE SCI FDN	1
SECOND HOSP JILIN UNIV	1
SECOND HOSP TANGSHAN	1
SECOND PEOPLES HOSP LIAO CHENG	1
SEIKOH GIKEN CO LTD	1
SELF DEF FORCE HOSP	1
SENTRX ANIM CARE INC	1
SEOUL INST	1
SEOUL JS HOSP	1
SEOUL JUN REHABIL CLIN	1
SERICA TECHNOL INC	1
SFR LYON EST	1

SHAANXI UNIV CHINESE MED	1
SHAHID BEHESHTI UNIV MED SCI	1
SHANGHAI CRYOWISE MED TECHNOL CO LTD	1
SHANGHAI JIAOTONG UNIV AFFILIATED PEOPLES HOSP 6	1
SHANGHAI LIQUN HOSP	1
SHANGHAI NINTH PEOPLES HOSP	1
SHANGHAI NORMAL UNIV	1
SHANGHAI TENTH PEOPLES HOSP	1
SHANGHAI UNIV MED HLTH	1
SHANGHAI UNIV MED HLTH SCI	1
SHANTOU UNIV	1
SHANXI MED UNIV	1
SHEBA MED CTR	1
SHENZHEN CHILDRENS HOSP	1
SHENZHEN GUANGMING NEW DIST PEOPLES HOSP	1
SHENZHEN INST GERIATR	1
SHENZHEN LONGGANG INST STOMATOL	1
SHIHEZI UNIV	1
SHINSHU UNIV	1
SHIRLEY RYAN ABIL LAB	1
SHOWA UNIV	1
SHRINERS HOSP CHILDREN ST LOUIS	1
SIGMUND FREUD UNIV	1
SINGAPORE GEN HOSP	1
SIRM	1
SJTUSM	1
SKUAST	1
SKUAST KASHMIR	1
SMITH NEPHEW GRP	1
SO CALIF CTR SPORTS MED	1
SO CALIF ORTHOPAED INST	1
SO GEN HOSP	1
SO ILLINOIS UNIV	1
SOKENDAI GRAD UNIV ADV STUDIES	1
SONORAN ORTHOPAED TRAUMA SURG	1
SOONCHUNHYANG UNIV	1
SOUTH AUSTRALIAN HLTH MED RES INST	1
SOUTH CENT UNIV NATIONALITIES	1
SOUTH MARKET HLTH CTR	1
SOUTHERN CALIF ORTHOPED INST	1
SOUTHERN CALIF PERMANENT MED GRP	1
SOUTHMEAD GEN HOSP	1
SOUTHWEST MED UNIV	1
SPORTHOPAEDICUM BERLIN	1
SPORTPRAXIS PROF DR KARSTEN KNOBLOCH	1

SPORTS MED UTAH	1
SQUIRE SANDERS US LLP	1
ST ANDREA HOSP	1
ST ANDREWS CTR PLAST SURG BURNS	1
ST CATHERINE SPECIALTY HOSP	1
ST GEORGES UNIV	1
ST GEORGES UNIV LONDON	1
ST JAMES UNIV HOSP	1
ST LOUIS CHILDRENS HOSP	1
ST LOUIS UNIV HOSP	1
ST MARYS UNIV	1
ST MICHAELS HOSP	1
ST ORSOLA MARCELLO MALPIGHI HOSP	1
ST PAULS HOSP	1
ST VINCENT HOSP	1
ST VINCENTS HOSP MELBOURNE	1
STANFORD MIPS	1
STANFORD SCH MED	1
STANFORD UNIV HOSP CLIN	1
STATE UNIV CAMPINAS UNICAMP	1
STEM BIO TEK CORP	1
STEM CELLS SPIN	1
STEM TECH GRP	1
STEPPING HILL HOSP	1
STEVENS INST TECHNOL	1
STRYKER ORTHOPAED	1
SUICHANG PEOPLES HOSP	1
SUN ORTHOPED EVANGEL COMMUNITY HOSP	1
SUNGHYUNKWAN UNIV	1
SUNWAY UNIV	1
SUSSEX EQUINE HOSP	1
SUZHOU MUNICIPAL HOSP	1
SW RES INST	1
SWANSON SCH ENGN	1
SWINBURNE UNIV TECHNOL	1
SWISS FED INST TECHNOL EHT ZURICH	1
SWISS FED INST TECHNOL LAUSANNE EPFL	1
SWISS FED INST TECHNOL ZURICH ETHZ	1
SWISS PARAPLEG CTR	1
SYNTHASOME INC	1
SYSTEMIX	1
TAIAN CITY CENT HOSP	1
TAIPEI MEDIAL UNIV	1
TAISHAN MED COLL	1
TAIZHOU HOSP	1

TAMPERE UNIV TECHNOL	1
TANGSHAN VOCAT TECH COLL	1
TAOS ORTHOPAED INST	1
TAOYUAN GEN HOSP	1
TAUBMAN CTR	1
TAYLORS UNIV	1
TCD	1
TEAGASC FOOD RES CTR	1
TEAGASC RES CTR	1
TEC PHARMA SA	1
TECH UNIV DARMSTADT	1
TECH UNIV DENMARK	1
TECHION INST	1
TECHMED CTR	1
TEDA PUHUA INT HOSP	1
TEGO SCI INC	1
TEI EPIRUS	1
TEJASVINI HOSP	1
TENGZHOU CENT PEOPLES HOSP	1
TENRI UNIV	1
TEVA PHARMACEUT IND LTD	1
TEXAS ORTHOBIOL INST	1
TEXAS TECH UNIV	1
THE SCIENTIST	1
THOMAS JEFFERSON UNIV HOSP	1
TIERKLIN SCHONBUHL	1
TIGENIX	1
TIGENIX LTD	1
TISSUE ENGN LAB	1
TISSUE ENGN SIGNALING TES ONLUS	1
TISSUE TRANSFORMAT TECHNOL INC	1
TITAN SPINE INC	1
TOBB UNIV ECON TECHNOL	1
TOHO UNIV	1
TOKYO MED UNIV	1
TOKYO METROPOLITAN BOKUTOH HOSP	1
TOKYO UNIV SCI	1
TOR VERGATA UNIV ROME	1
TORANOMON GEN HOSP	1
TORONTO REHABIL INST UHN	1
TRABZON KANUNI TRAINING RES HOSP	1
TRADIT CHINESE MED HOSP	1
TRADIT CHINESE MED SHAOXING HOSP	1
TRANSLAT CTR REGENERAT MED	1
TRANSTISSUE TECHNOL GMBH	1

TRANSTISSUE TECHNOL GMBH BERLIN	1
TRENTO RES UNIT	1
TRIINST TRAINING PROGRAM COMPUTAT BIOL MED	1
TRINITY SPORTS MED PERFORMANCE CTR	1
TRISAKTI UNIV	1
TRISTATE SPINE CARE INST	1
TSURUMI UNIV	1
TULANE SCH MED	1
TYNDALL NATL INST	1
UAB	1
UC BIOTECH CANTANHEDE	1
UCL INST ORTHOPAED MUSCULOSKELETAL SCI	1
UCL INST ORTHOPAED MUSCULOSKELETAL SCI IOMS	1
UCL ROYAL FREE UNIV COLL LONDON	1
UCLA	1
UCLH	1
UCONN HLTH CTR	1
UDELAR	1
UIT ARCTIC UNIV NORWAY	1
ULM UNIV	1
UMR CNRS 5246	1
UNESP JABOTICABAL	1
UNESP SAO PAULO STATE UNIV	1
UNIARARAS	1
UNIDAD CIRUGIA ARTROSCOP MIKEL SANCHEZ	1
UNIKLIN BALGRIST	1
UNION MEM HOSP	1
UNIV AALBORG	1
UNIV ABERDEEN	1
UNIV AFFILIATED SOUTHWEST HOSP	1
UNIV APPL SCI	1
UNIV ARKANSAS MED SCI	1
UNIV AUTONOMA AGUASCALIENTES	1
UNIV AUTONOMA SAN LUIS POTOSI	1
UNIV AVEIRO	1
UNIV BERGAMO	1
UNIV BERGEN	1
UNIV BOLOGNA ALMA MATER STUDIORUM	1
UNIV BONN	1
UNIV BRASILIA	1
UNIV BRASILIA UNB	1
UNIV BRESCIA	1
UNIV BRISTOL	1
UNIV CALIF BERKELEY CHONGQING UNIV JOINT PHD STUD	1
UNIV CAMPINAS UNICAMP	1

UNIV CAMPUS BIOMED ROMA	1
UNIV CAMPUS BIOMEDICO ROMA	1
UNIV CANTABRIA IFIMAV	1
UNIV CAPE TOWN	1
UNIV CASTELO BRANCO	1
UNIV CATANIA	1
UNIV CATOLICA	1
UNIV CATOLICA BRASILIA	1
UNIV CATOLICA DOM BOSCO	1
UNIV CATOLICA MURCIA	1
UNIV CATOLICA TEMUCO	1
UNIV CATOLICA VALENCIA SAN VICENTE MARTIR	1
UNIV CHILDRENS HOSP	1
UNIV CHILE	1
UNIV CHINESE ACAD SCI	1
UNIV CLIN CARL GUSTAV CARUS DRESDEN	1
UNIV CLIN DRESDEN	1
UNIV CLIN ORTHOPAED	1
UNIV CLIN TRAUMA SURG SPORTS INJURIES	1
UNIV COLL HOSP GALWAY	1
UNIV COLL LONDON UCL	1
UNIV CORDOBA	1
UNIV CRETE	1
UNIV CTR HERMINIO OMETTO FDN FHO	1
UNIV CUIABA	1
UNIV DUBLIN TRINITY COLL	1
UNIV ENVIRONM LIFE SCI	1
UNIV ENVIRONM LIFE SCI WROCLAW	1
UNIV ESTADUAL MARINGA	1
UNIV ESTADUAL SANTA CRUZ	1
UNIV ESTADUAL SAO PAULO	1
UNIV FED MATO GROSSO DO SUL	1
UNIV FED MINAS GERAIS UFMG	1
UNIV FED RIO DE JANEIRO	1
UNIV FED RIO GRANDE DO SUL	1
UNIV FED RIO GRANDE SUL UFRGS	1
UNIV FED SANTA CATARINA	1
UNIV FED SAO CARLOS	1
UNIV FED VALE DO JEQUITINHONHA MUCURI	1
UNIV FEDERICO II NAPLES	1
UNIV FREIBURG	1
UNIV FRIBOURG	1
UNIV FUKUI	1
UNIV G DANNUNZIO FDN	1
UNIV GADJAH MADA	1

UNIV GENOA	1
UNIV GREIFSWALD	1
UNIV GRENOBLE ALPES	1
UNIV HALLE WITTENBERG	1
UNIV HAMBURG	1
UNIV HARTFORD	1
UNIV HENRI POINCARE	1
UNIV HLTH NETWORK	1
UNIV HOSP 1	1
UNIV HOSP AUGSBURG	1
UNIV HOSP BERN	1
UNIV HOSP CLEVELAND	1
UNIV HOSP CTR SESTRE MILOSRDNICE	1
UNIV HOSP JENA	1
UNIV HOSP LAUSANNE CHUV	1
UNIV HOSP RWTH AACHEN	1
UNIV HOSP SAO PAULO	1
UNIV HOSP SCHLESWIG HOLSTEIN	1
UNIV HOSP SVETI DUH	1
UNIV HOSP ULM	1
UNIV HOSP VIRGEN VICTORIA	1
UNIV HOSP WURZBURG	1
UNIV HULL	1
UNIV IDAHO	1
UNIV INNSBRUCK HOSP	1
UNIV INSUBRIA	1
UNIV INT CATALUNYA	1
UNIV JENA	1
UNIV KLINIKUM REGENSBURG	1
UNIV LA FRONTERA	1
UNIV LA LAGUNA	1
UNIV LA SAPIENZA BIOMED SCI PK SAN RAFFAELE	1
UNIV LAGOS	1
UNIV LAHORE	1
UNIV LAUSANNE	1
UNIV LAVAL	1
UNIV LEICESTER	1
UNIV LEON	1
UNIV LIEGE	1
UNIV LIFE SCI LUBLIN	1
UNIV LILLE NORD FRANCE	1
UNIV LITTORAL COTE DOPALE	1
UNIV LIVERPOO	1
UNIV LJUBLJANA	1
UNIV LOUISVILLE	1

UNIV LUND	1
UNIV LUND HOSP	1
UNIV MANSOURA	1
UNIV MASSACHUSETTS	1
UNIV MED BERLIN	1
UNIV MED CTR HAMBURG EPPENDORF	1
UNIV MED CTR ROTTERDAM	1
UNIV MED PHARM CRAIOVA	1
UNIV MESSINA	1
UNIV MICHIGAN SURG	1
UNIV MISSISSIPPI	1
UNIV MIYAZAKI	1
UNIV MONTPELLIER 2	1
UNIV MONTREAL HOSP	1
UNIV MYSORE	1
UNIV NANCY 1	1
UNIV NAT RESOURCES LIFE SCI	1
UNIV NEW MEXICO	1
UNIV NICE	1
UNIV NORTH CAROLINA CHARLOTTE	1
UNIV NORTH TEXAS	1
UNIV NOVA LISBOA	1
UNIV NOVE JULHO UNINOVE	1
UNIV OCCUPAT ENVIRONM HLTH	1
UNIV OSIJEK	1
UNIV OSLO	1
UNIV OTAGO	1
UNIV PARIS 05	1
UNIV PARIS DIDEROT	1
UNIV PARIS EST CRETEIL	1
UNIV PARIS SACLAY	1
UNIV PARIS SUD	1
UNIV PAUIISTA	1
UNIV PICARDIE JULES VERNE	1
UNIV PIEMONTE ORIENTALE	1
UNIV PITTSBURGH MED CTR UPMC HAMOT	1
UNIV POLITECN VALENCIA	1
UNIV PUERTO RICO	1
UNIV REIMS	1
UNIV RIJEKA	1
UNIV ROME	1
UNIV ROSTOCK	1
UNIV S DAKOTA	1
UNIV S MANCHESTER HOSP	1
UNIV SALENTO	1

UNIV SALZBURG	1
UNIV SANTO TOMAS	1
UNIV SCI PHILADELPHIA	1
UNIV SEBELAS MARET	1
UNIV SIEGEN	1
UNIV SIENA	1
UNIV SOUTH AUSTRALIA	1
UNIV SOUTH CAROLINA	1
UNIV SPITAL ZURICH	1
UNIV ST ANDREWS	1
UNIV STATE SAO PAULO	1
UNIV STRATHCLYDE	1
UNIV SUSSEX	1
UNIV TASMANIA	1
UNIV TECHNOL	1
UNIV TEKNOL MALAYSIA	1
UNIV TEXAS HLTH SCI CTR HOUSTON UT HLTH	1
UNIV TEXAS MD ANDERSON CANC CTR	1
UNIV TEXAS SOUTHWESTERN MED CTR	1
UNIV THESSALY	1
UNIV TOKUSHIMA	1
UNIV TOKYO HOSP	1
UNIV TRAS OS MONTES ALTO DOURO	1
UNIV TSUKUBA	1
UNIV TUBINGEN HOSP	1
UNIV URBINO CARLO BO	1
UNIV VALLADOLID	1
UNIV VEIGA ALMEIDA	1
UNIV VERONA ITALY	1
UNIV VET ANIM SCI	1
UNIV VET MED	1
UNIV VET PHARMACEUT SCI BRNO	1
UNIV WARSAW	1
UNIV WARWICK	1
UNIV WEST INDIES	1
UNIV WITTEN HERDECKE	1
UNIV WOLLONGONG	1
UNIV ZAGREB	1
UNIV ZURICH BALGRIST	1
UPMC CTR SPORTS MED	1
US ARMY INST SURG RES	1
US DEPT COMMERCE	1
USC KECK SCH MED	1
USN	1
USP HOSP	1

VA BOSTON HEALTHCARE SYST	1
VA NEBRASKA WESTERN IOWA HLTH CARE S	1
VALLEY ANESTHESIOL PAIN CONSULTANTS ENVIS PHYS	1
VANCOUVER COASTAL HLTH RES INST	1
VANDERBILT UNIV	1
VARINOR MAT SA	1
VET ACTIV LTDA	1
VET ADM MED CTR	1
VET ADM PALO ALTO	1
VET ADM PALO ALTO HEALTHCARE SYST	1
VET AFFAIRS HOSP	1
VET AFFAIRS PALO HLTH CARE SYST	1
VET AFFAIRS SAN DIEGO HEALTHCARE SYST	1
VET CLIN	1
VET ORTHOPED SPORTS MED	1
VET ORTHOPED SPORTS MED GRP	1
VET ORTHOPED SURG SERV	1
VET STUDIO	1
VETCELL BIOSCI	1
VETMEDUNI VIENNA	1
VICTOR BABES UNIV MED PHARM TIMISOARA	1
VICTORIAN NODE AUSTRALIAN NATL FABRICAT	1
VICTORIAN NODE AUSTRALIAN NATL FABRICAT FACIL	1
VIENNA VET SCH	1
VIRGINIA MARYLAND COLL VET MED	1
VIRGINIA MARYLAND REG COLL VETERI MED	1
VIRTUAL UNIV PAKISTAN	1
VIT	1
VITEC VIDEOCOM LTD	1
VORNIA BIOMAT	1
VRIJE UNIV AMSTERDAM	1
VRIJE UNIV BRUSSEL	1
WAKE FOREST UNIV HLTH SCI	1
WALTER REED NATL MIL MED CTR	1
WALTHAM CTR PET NUTR	1
WARSAW MED UNIV	1
WASEDA UNIV	1
WASHINGTON UNIV ST LOUIS	1
WEILL CORNELL MED SCH	1
WEIZMANN INST SCI	1
WELLCOME TRUST CTR CELL MATRIX RES	1
WENZHOU CENT HOSP	1
WEST VIRGINIA UNIV	1
WESTERN NEW ENGLAND UNIV	1
WHISTON GEN HOSP	1

WHISTON HOSP	1
WISTAR INST ANAT BIOL	1
WROCLAW RES CTR ETT	1
WROCLAW UNIV ENVIMNMCNTAL LIFE SCI	1
WUHAN GEN HOSP GUANGZHOU COMMAND	1
WUHAN GEN HOSP GUANGZHOU MIL COMMAND	1
WUHAN TEXT UNIV	1
WYETH RES	1
XIAN MED UNIV	1
XIANGFAN CENT HOSP	1
XINXIANG MED COLL	1
XUZHOU MATERN CHILD HLTH CARE HOSP	1
YANCHENG INST TECHNOL	1
YESHIVA UNIV	1
YILDIZ TECH UNIV	1
YOKOHAMA CITY UNIV	1
YOKOHAMA UNIV PHARM	1
YOKUSHUKAI DENT CLIN	1
YONSEI SARANG HOSP	1
YUE BEI PEOPLES HOSP	1
YUKIOKA HOSP	1
ZAND INST HIGHER EDUC	1
ZHEJIANG PROV KEY LAB ORTHOPAED	1
ZHEJIANG PROV PEOPLES HOSP	1
ZHEJIANG SCI TECH UNIV	1
ZHEJIANG XINGYUE BIOTECHNOL CO LTD	1
ZIMMER INC	1
ZONGULDAK KARAELMAS UNIV	1
ZURICH UNIV APPL SCI	1
ZYMOGENET INC	1
