# Supplementary material for: Global Research Trends in Tendon Stem Cells from 1991 to 2020: A Bibliometric and Visualized Study
Source: Stem Cells Int. 2022 Jun 18;2022:7937765. doi: 10.1155/2022/7937765 (PMC9233735; doi:10.1155/2022/7937765)
Supplement: Supplementary 4 — Supplementary Table 4: the five clusters of keywords on tendon stem cells research from 1991 to 2020. Keywords with minimum occurrences over 20 were included in the cooccurrence analysis. [file 7937765.f4.pdf]

Supplementary Table 4. The five clusters of keywords on tendon stem cells research from 1991 to 2020

Cluster 1: Animal study (54)	Cluster 2: Tissue engineering (39)	Cluster 3: Clinical study (30)	Cluster 4: Mechanism research (50)	Cluster 5: Stem cells research (40)
acl reconstruction	adhesion	achilles tendinopathy	activation	adipose tissue
allograft	alignment	achilles tendon	age-related-changes	adipose-tissue
animal model	biocompatibility	achilles-tendon	apoptosis	articular-cartilage
animal-models	biomaterials	achilles-tendon	connective-tissue	autologous chondrocyte implantation
anterior cruciate ligament	bioreactor	basic science	cytokines	bone marrow
arthroscopic repair	bone	biology	differentiation	bone regeneration
augmentation	cells	delivery	endothelial-cells	bone-marrow
autograft	collagen	double-blind	expression	cartilage
biomechanical properties	collagen sponge constructs	endothelial growth-factor	extracellular matrix	cell therapy
biomechanics	constructs	flexor tendon	extracellular-matrix	chondrocytes
bone morphogenetic protein-2	cross-linking	gene therapy	fibroblasts	chondrogenesis
cruciate ligament reconstruction	degradation	gene-transfer	gene	chondrogenic differentiation
defect	electrospinning	growth factor	gene expression	culture
degeneration	engineered tendon	growth factors	gene-expression	defects
enhancement	fabrication	growth-factor-i	growth	digital flexor tendon
enthesis	fibers	growth-factors	identification	equine

fatty infiltration	fibroblast-growth-factor	healing	inflammation	horse
follow-up	flexor tendons	heterotopic ossification	injury	horses
graft	hydrogel	injection	matrix	human adipose-tissue
grafts	in-vitro	management	mechanisms	human bone-marrow
growth-factor	ligament	pathogenesis	messenger-rna	implantation
growth-factor-beta	mechanical stimulation	platelet-rich plasma	mice	injuries
in-vivo	mechanical-properties	prp	migration	intraarticular injection
insertion	mechanobiology	rabbit achilles-tendon	muscle	marrow stromal cells
integrity	mechanotransduction	randomized controlled-trial	osteoblasts	mesenchymal stem cell
knee	nanofiber scaffolds	repair	osteogenic differentiation	mesenchymal stem cells
marrow	nanofibers	tendinopathy	phenotype	mesenchymal stromal cells
medial collateral ligament	nanofibrous scaffolds	tendinosis	progenitors	osteoarthritis
mesenchymal stem-cells	scaffold	tendon healing	proliferation	osteogenesis
model	scaffolds	tendon injury	promotes	progenitor cells
morphogenetic protein-2	silk scaffold		protein	regenerative medicine
outcomes	tendon		rat	stem cell
patellar tendon	tendon tissue engineering		responses	stem cells
rabbit	tenogenic differentiation		scleraxis	stromal cells
rabbit model	tissue		scleraxis expression	tendinitis
rat model	tissue engineering		self-renewal	tendon injuries
reconstruction	tissue regeneration		stem-cells	tendon repair

regeneration	vitro	stem/progenitor cells	therapy
rotator cuff	vivo	stimulation	transplantation
rotator cuff repair		tenascin-c	umbilical-cord blood
rotator cuff tear		tendon differentiation	
rotator cuff tears		tendon regeneration	
rupture		tendon stem cells	
satellite cells		tendon stem/progenitor cells	
shoulder		tendon-derived stem cells	
skeletal-muscle		tendons	
small-intestinal submucosa		tenocyte	
supraspinatus		tenocytes	
supraspinatus tendon		tenomodulin	
surgical repair		tgf-beta	
tears			
tendon graft			
tendon-bone healing			
tunnel			
